# Forward Genetic Analysis to Identify Determinants of Dopamine Signaling in *Caenorhabditis elegans* Using Swimming-Induced Paralysis

**DOI:** 10.1534/g3.112.003533

**Published:** 2012-08-01

**Authors:** J. Andrew Hardaway, Shannon L. Hardie, Sarah M. Whitaker, Sarah R. Baas, Bing Zhang, Daniel P. Bermingham, Ariana J. Lichtenstein, Randy D. Blakely

**Affiliations:** * Department of Pharmacology; † Department of Biomedical Informatics; ‡ Silvio O. Conte Center for Neuroscience Research, and; § Department of Psychiatry, Vanderbilt University School of Medicine, Nashville, Tennessee 37232-8548

**Keywords:** presynaptic, dopamine, transporter, receptor, *Caenorhabditis elegans*, forward genetics

## Abstract

Disrupted dopamine (DA) signaling is believed to contribute to the core features of multiple neuropsychiatric and neurodegenerative disorders. Essential features of DA neurotransmission are conserved in the nematode *Caenorhabditis elegans*, providing us with an opportunity to implement forward genetic approaches that may reveal novel, *in vivo* regulators of DA signaling. Previously, we identified a robust phenotype, termed Swimming-induced paralysis (Swip), that emerges in animals deficient in the plasma membrane DA transporter. Here, we report the use and quantitative analysis of Swip in the identification of mutant genes that control DA signaling. Two lines captured in our screen (*vt21* and *vt22)* bear novel *dat-1* alleles that disrupt expression and surface trafficking of transporter proteins *in vitro* and *in vivo*. Two additional lines, *vt25* and *vt29*, lack transporter mutations but exhibit genetic, biochemical, and behavioral phenotypes consistent with distinct perturbations of DA signaling. Our studies validate the utility of the Swip screen, demonstrate the functional relevance of DA transporter structural elements, and reveal novel genomic loci that encode regulators of DA signaling.

The catecholamine dopamine (DA) is a phylogenetically conserved neurotransmitter that in vertebrates, including humans, regulates motor and cognitive behavior. Altered DA signaling contributes to several disorders of the brain, including Parkinson disease, dystonia, attention-deficit/hyperactivity disorder, schizophrenia, and addiction (Carlsson 1993; Mazei-Robison *et al.* 2005; Seeman 2010; Kurian *et al.* 2011). DA signaling is achieved through both presynaptic and postsynaptic mechanisms that, to date, have been studied largely using biochemical, pharmacological, and reverse genetic approaches. These studies have uncovered and characterized many fundamental components that control DA signaling, such as the genes that provide for DA synthesis, release, reuptake, and response. The powerful, modulatory control exerted by DA over both cognitive and motor behavior and the incomplete understanding of the determinants of risk for DA-associated brain disorders suggest that a broader array of genes exists that controls DA signaling. The identification of these genes may benefit therefore from unbiased approaches, such as those afforded by forward genetic screens.

Among the most critical of known determinants of DA signaling, one molecule, the presynaptic DA transporter (DAT; *SLC6A3*), has special roles in controlling access of pre- and postsynaptic DA receptors to DA, in recycling DA into presynaptic terminals after release, and in maintaining levels of DA needed for sustained release (Giros *et al.* 1996; Torres *et al.* 2003). The knockout of DAT through homologous recombination in mice demonstrates an obligate role for the transporter in amphetamine and cocaine-induced hyperlocomotion, as well as DA release and clearance (Giros *et al.* 1996). Conversely, mice overexpressing DAT display reduced extracellular DA levels and heightened sensitivity to amphetamine (Salahpour *et al.* 2008). These studies also demonstrate that genetic manipulation of DAT in mice leads to changes in the expression of genes that encode pre- and postsynaptic DA receptors and neuropeptides, underscoring the importance of DAT as a key regulator of a broad DA signaling network (Giros *et al.* 1996). After the cloning of DAT cDNAs (Kilty *et al.* 1991; Shimada *et al.* 1991; Giros *et al.* 1992; Jayanthi *et al.* 1998; Brüss *et al.* 1999), many have explored the impact of DAT mutations using heterologous expression models *in vitro* (Schmitt and Reith 2010), although, as of yet, the significance *in vivo* of many of these findings is unknown. Recently, we and others have identified rare, functionally penetrant, mutations in DAT in subjects with attention-deficit/hyperactivity disorder and juvenile dystonia (Mazei-Robison *et al.* 2008; Kurian *et al.* 2009), compelling a better understanding of the impact of DAT mutations and altered DAT regulatory mechanisms *in vivo*.

Forward genetic strategies that rely on DAT-dependent phenotypes provide a path to the identification of key DAT structural elements as well as the elucidation of novel regulators of DA signaling. Although such methods overcome the bias of studies focused on known genes and pathways (Mohn *et al.* 2004), they are typically quite difficult to implement in mammals because of the time and cost associated with animal breeding, mutation mapping, and functional characterization *in vivo*. The nematode *Caenorhabditis elegans* has long been used in forward genetic screens (Brenner 1974), including identification of genes supporting DA signaling (Sulston *et al.* 1975; Schafer and Kenyon 1995; Sawin *et al.* 2000; Chase *et al.* 2004; Allen *et al.* 2011; Wani *et al.* 2012). These and homology-based approaches have revealed *C. elegans* orthologs of mammalian genes required for DA biosynthesis, including tyrosine hydroxylase/*cat-2* (Lints and Emmons 1999), GTP cyclohydrolase/*cat-4* (Loer and Kenyon 1993), and amino acid decarboxylase/*bas*-1 (Bamford *et al.* 2004), the vesicular monoamine transporters/*cat-1* (Duerr *et al.* 1999), genes involved in DA response including the D1 and D2 type DA receptors *dop-1/2/3/4* (Suo *et al.* 2003; Chase *et al.* 2004; Suo *et al.* 2004; Sugiura *et al.* 2005), and the presynaptic DA transporter/*dat-1* (Jayanthi *et al.* 1998).

In a previous study, we demonstrated that worms lacking functional DAT-1 (*dat-1(ok157)*) demonstrate a robust, DA-dependent phenotype when placed in water, termed Swimming-induced paralysis (Swip), a behavior that is dependent on activation of the postsynaptic, D2-like receptor DOP-3 (McDonald *et al.* 2007). Here, we describe our efforts to capitalize on the Swip phenotype to capture modulators of endogenous DA signaling using forward genetic approaches. Lines derived from this effort are subjected to conventional biochemical and pharmacological methods, as well as to a novel analytical platform that can more finely dissect features of disrupted motor function. We describe the isolation of multiple, independent mutant lines, including two (*vt21* and *vt22*) that possess novel alleles of DAT-1. Disrupted expression and trafficking of the *vt21* mutant provides the first demonstration of functional relevance *in vivo* of a highly conserved, structural feature of SLC6 transporters. Two additional lines, *vt25* and *vt29*, lack *dat-1* mutations, map to different genomic loci, and sustain distinct components of DA signaling.

## Materials and Methods

### *C. elegans* strains, husbandry, and genotyping

*C. elegans* strains were cultured on bacterial lawns of OP50 and maintained at 12 to 20° using standard methods (Brenner 1974) unless otherwise noted. The wild-type strain is N2 Bristol. The *dat-1(ok157)III* strain was a gift of J. Duerr and J. Rand (Oklahoma Medical Research Foundation, Oklahoma City) and is a complete loss-of-function mutation that eliminates the majority of the DAT-1 coding sequence. BY200 and BY250 are stable integrants (Nass *et al.* 2002) that express the transcriptional fusion P*_dat-1_*::GFP (pRB490) on an N2 background. BY250 (*vtIs7*) was used for imaging DA neuron morphology of cloned and outcrossed *swip* lines and for 6-hydroxydopamine (6-OHDA) experiments. A line producing a loss-of-function disruption of DOP-3 (*dop-3(vs106)X*) was obtained from the *Caenorhabditis* Genetics Center (University of Minnesota, Minneapolis) and *cat-2(tm2261)* from Shohei Mitani at the National Bioresource Project at the Tokyo Women's Medical University. *C. elegans* genomic DNA was isolated as described previously (Nass *et al.* 2005) and used at a concentration of 1 ng/μL to genotype lines by polymerase chain reaction (PCR). For all crosses, either males were generated using the method originally described by Sulston and Hodgkin (Hodgkin 2005), or alleles were crossed to males of publically available strains containing integrated fluorescent transgenes that mark distinct nematode structures. After 24 hr of mating, hermaphrodites were separated onto individual plates and considered successful if ~50% of the progeny from an individual hermaphrodite are males and/or if the F1 progeny contained the fluorescent transgene from the male parent. For generation of the *dat-1(ok157);vt29* strain, markers of LGIII and LGX were used, and the double-mutant genotype was confirmed by resegregation of the two genes and confirmation with PCR genotyping of *ok157* and SWIP testing for *vt29*. Generation of *vt25,dat-1(ok157)* will require recombination of the two alleles after identification of the *vt25* functional variant and will be presented in a later report.

### *C. elegans* assay for Swip

In both batch and automated analyses, we generated synchronous populations of these strains by hypochlorite treatment and harvesting arrested L1 animals. Early- to mid-stage L4 animals were identified by characteristic morphology and used for behavior because N2 animals show some stochastic Swip and quiescence bouts during the last larval molt. For automated analysis, single L4 hermaphrodites were placed in 20 μL of water in a single well of a Pyrex Spot Plate (Fisher; cat. no. 13-748B), and 10-min movies (uncompressed AVI format) of their swimming behavior were created and analyzed as described previously (Matthies *et al.* 2006; McDonald *et al.* 2007), with slight modifications.

We processed thrashing data using an in-house movement processing program (Worm Tracker, available on request) that fits a five-point spine to the worm in each frame. The five-point spines consist of four segments and three “joints.” In Worm Tracker, a spine is specified by the x and y positions of the spine center, the rotation of the spine with respect to the vertical axis, and finally the angles of the three joints. The spine positions are fit using a particle filter and motion detection. The Worm Tracker software processes a video and records the position of the fitted spine for each frame. A MATLAB script reads the exported files and computes the frequency of swimming using fast Fourier transform and by counting frames between angular extrema. This script then produces a data file that provides the frequency of motion of a given worm over time. These individual files are then grouped by genotype into a large data matrix and an accompanying annotation file made for each of the animals to be analyzed. These two files are then used as the input for a custom script written in the free publically available statistical program R. The program (SwimR, available upon request) smoothens individual data traces using a specified moving window and identifies any outliers in the sample file using a modified z-score calculation (Iglewicz and Hoaglin 1993). SwimR then generates several output text and PDF files, including a scatterplot of average frequency *vs.* time, a heat map of the samples ordered by strength of paralysis, and a histogram of the binned frequency data. For paralyzers, the script calculates the latency to paralyze and several parameters that define the ability of individual animals to revert from paralysis to regular thrashing activity, including reversion probability (total time in reversion/total time after paralysis), time to first reversion, average reversion duration, and reversion strength (area under the curve during all reversion events). Parameters related to paralysis can be set by the user.

We defined paralyzed animals in our studies as those animals that fall below 20% of their maximal thrashing value and stay below this threshold for at least 20 sec. Revertants are defined as those animals that, after paralyzing, recross a threshold equivalent to 50% of their maximum thrashing rate for any length of time. For batch analysis, 10 to 20 worms were visually scored in a single well of the Pyrex Spot Plate. Worms displaying Swip after a 10-min assay period were counted and in some cases isolated by hand for further tests. For reserpine treatments, worms from each line were first synchronized by hypochlorite treatment, and L1 larvae are grown on OP50 plates containing 0.6 mM reserpine. After ~48 hr of reserpine or vehicle treatment, the population Swip analysis was repeated on groups of L4 animals from each line.

### Exogenous DA sensitivity assay

Assays were performed as described in Chase *et al.* 2004, except that L4 animals were used in lieu of young adults because this stage is most relevant to the DAT-1−dependent Swip phenotype. To summarize, 10 L4 animals were transferred to 1.7% agar plates containing 2 mM glacial acetic acid and various concentrations of DA, incubated for 20 min, and then scored as paralyzed or moving. Animals were defined as moving if they were able to propagate a body bend through a minimum or maximum amplitude. All concentrations of DA were used on the same day.

### *C. elegans* mutagenesis screen

Standard methods for a nonclonal, F2 screen were performed as originally described (Brenner 1974) and were used on wild-type hermaphrodite worms carrying a DAT-1 promoter-driven green fluorescent protein (GFP) transgene (BY200) (Nass *et al.* 2002). A semisynchronous population of healthy, well-fed late L4 animals was exposed to either 47 mM ethylmethanesulfonate (EMS) or 0.5 mM *N*-ethyl-*N*-nitrosourea at room temperature for 4 hr in a chemical fume hood. After 24 hr of recovery, 30 gravid adult worms were placed on each of eight 10-cm OP50 plates and allowed to lay ~50 eggs each (the F1s) before being discarded. After reaching adulthood and laying 20 to 30 eggs each for a total of 1000 to 1500 developing F2 animals per plate, the F1 animals were discarded. When F2 animals reached the L4 stage, they were batch screened for the Swip phenotype by rinsing off the plate and analyzing 50 to 100 animals per well as described previously. We tracked the source plate of each F2 so that only one stable mutant line was kept for each plate of the mutant F1s. After 10 min, animals that exhibited Swip were replated and allowed to recover. Swip-positive animals that recovered normal movement on solid media were cloned and retested in Swip assays to establish phenotype stability. Only lines in which at least 50% of the animals displayed Swip on retest were saved for a test of reserpine reversal of Swip, as described previously. In later rounds of screening, this convention was increased to 80% to improve recovery of stable lines. Stable lines that demonstrated a significant rescue of Swip after reserpine treatment were kept for further analysis. All recovered lines passing the reserpine test were outcrossed to the N2 strain a minimum of three times before further analysis. After each outcross, lines were rehomozygosed and retested for stable Swip on separate days with multiple parental founders before proceeding to the next cross. All lines recovered from the screen were sequenced with sense and antisense primers that span all DAT-1 exons and includes 1 kb upstream of the transcription start site as well as 50 bp downstream of the translational stop codon) using Big Dye Terminator Cycle Sequencing Mix (ABI, Foster City, CA). PCR products were sequenced on an ABI 3730xl DNA Analyzer (DNA Sequencing Core Facility, Vanderbilt Division of Medicine).

### SNP mapping

Mapping of mutant loci was performed as described previously (Davis *et al.* 2005). In summary, stable outcrossed *swip* strains were crossed to the CB4856 strain. For bulk segregant analysis, lysates from both Swip-positive and Swip-negative F2 populations were generated and used as the input for genome-wide, 96-well PCR. N2 animals were not used as a control in these efforts because we found nonspecific N2 Bristol islands in both the Swip-positive and Swip-negative F2s on the left arm of LGI. The bulk segregant protocol was used to identify linkage groups that can serve for fine mapping with experiments replicated at least twice with separate populations to demonstrate consistent linkage. For fine-interval mapping, individual Swip-positive F2s were cloned, and their F3 progeny were tested for a stable Swip phenotype. Mutations were considered homozygous if the F3 population demonstrated Swip comparable to the original strain. Populations were manually scored in at least four to five assays using 40 to 50 worms. DNA from individual clones was then used as the input for PCR of individual intervals to ascertain a specific Bristol island on the mapped linkage group.

### Creation of plasmids and transgenic animals

#### Plasmids:

P*_dat-1_*:GFP::DAT-1(Carvelli *et al.* 2004) and P*_dat-1_*::GFP (Nass *et al.* 2002) have been described previously. The following plasmids were created to examine DAT-1 mutations in heterologous mammalian expression systems: pRB1026 [DAT-1(G460D) in pcDNA3]; pRB1027 [DAT-1(W283^*^) in pcDNA3]; pRB1028 [N-terminally HA-tagged DAT-1(G460D) in pcDNA3]; and pRB1029 [N-terminally HA-tagged DAT-1 (W283^*^] in pcDNA3) were developed to examine the DAT-1 mutations *vt21* (G460D) and *vt22* (W283^*^). pRB1030 [DAT-1(G460D) in pRB491] and pRB1031 [DAT-1(W283^*^) in pRB491] were created to evaluate the DAT-1 mutations *vt21* (G460D) and *vt22* (W283^*^) alleles in *C. elegans in vivo*. Lines shown in Figure 2 are *ex21*, *ex62*, and *ex65* for DAT-1, *vt21*, and *vt22*. All mutations were created using the Quick Change XL site-directed mutagenesis kit (Stratagene, La Jolla, CA).

#### Transgenic animals:

Fully sequenced constructs (90 ng/μL) were coinjected with the lin-15 rescuing plasmid pJM23 (40 ng/μL) into *dat-1(ok157);lin-15(n765ts)* animals using methods described previously (Jin *et al.* 1999). *vtIs18* is an outcrossed integrant of *ex21* that expresses a P*_dat-1_*:GFP::DAT-1, rescues the *dat-1* strain, and maps to LGV.

### Mammalian cell culture and western blot analysis

Methods for western blot analysis and surface biotinylation of the *C. elegans* DAT-1 expressed in COS-7 have been described elsewhere (Carvelli *et al.* 2004; Nass *et al.* 2005). In summary, COS-7 cells were plated and allowed to attach for 24 hr before transfection. Cells were transfected with 250 ng of either HA-tagged DAT-1 cDNA (pRB606) (Jayanthi *et al.* 1998), HA-tagged DAT-1(G460D) cDNA (*vt21*, pRB1028), HA-tagged DAT-1(W283^*^) cDNA (*vt22*, pRB1029), or an empty vector (pcDNA3; Invitrogen, Carlsbad, CA) using TransIT-LT1 (Mirus, Madison, WI) as the transfection vehicle. At 48 hr after transfection, cells were harvested as previously described, and equal amounts of sample protein were resolved on a 4%–20% TRIS SDS-Page Ready Gel (Bio-Rad; cat. no. 161-1105) followed by transfer overnight to an Immobilon-P PVDF membrane (Millipore; cat. no. IPVH00010). Membranes were blocked for 1 hr in phosphate-buffered saline containing 0.5% TWEEN20 and 5% instant nonfat dried milk. After blocking, the membranes were incubated with a rat monoclonal anti-HA-peroxidase labeled antibody [High Affinity (3F10), Roche Diagnostics] at a 1:2000 dilution for 1 hr at room temperature. After being washed, blots were developed with Western Lightening chemiluminescence reagents (Perkin Elmer) and exposed to High Performance Chemiluminescence film (Amersham, Piscataway, NJ). To analyze surface expression, cells were biotinylated with sulfosuccinimidyl-2-(biotinamido)ethyl-1,3-dithiopropionate-(sulfo-NHS-SS-Biotin; 1.0 mg/mL; Pierce, Rockford, IL) and after detergent extraction, samples were incubated with Streptavidin beads and eluted for SDS-PAGE analysis as previously described (Nass *et al.* 2005).

### *In vivo* GFP imaging

*In vivo* imaging of DAT-GFP fusion protein expression and distribution was performed as previously described (McDonald *et al.* 2007). To summarize, a series of 1.2-μm image planes was obtained using a Zeiss LSM 510, creating a “Z stack.” Because of differences in GFP expression, different laser settings were used for imaging of the head neurons (CEP and ADE) and PDE neurons. Higher laser settings also are used to detect the GFP tagged *vt22* DAT variant. Z stacks were used to create 3D reconstructions of images as noted in the figure legends. Image stacks containing either cell body or synaptic regions were thresholded, rank filtered, and pseudocolored using Metamorph (Molecular Devices, Sunnyvale, CA). Final images of either CEPs and ADEs or PDEs were processed using identical brightness and contrast values.

### HPLC measurement of DA levels

Synchronized L4 worms were washed in water, pelleted, frozen, and then sonicated in buffer containing EDTA as previously described (Benedetto *et al.* 2010). Protein content was measured by a BCA assay (Pierce) using 10 μL of the lysate. Then, 5 ng/mL isoproterenol was added to the remaining sample, which was then applied to an alumina column to extract DA. Eluted samples were then analyzed by HPLC using a Waters 717+ autosampler and an Antec Decade II electrochemical detector (oxidation: 0.5V). Reverse-phase high-performance liquid chromatography (HPLC) was performed using a Phenomenex Nucleosil (5 μm, 100 A) C18 column (150 × 4.60 mm) with a mobile phase of 89.5% 0.1 M TCA, 0.1 M sodium acetate, 1 mM EDTA, and 10.5% methanol (pH 3.8) at a rate of 0.6 mL/min, with data acquired by Millennium 32 software.

### 6-OHDA toxicity assay

DA neuron sensitivity to 6-OHDA was assessed as described previously (Nass *et al.* 2002, 2005; McDonald *et al.* 2007). In brief, strains containing the *vtIs7* (p*_dat-1_*:GFP) transgene were grown on NA22 media containing plates seeded with 8P bacteria (8P/NA22) and synchronized at the L1 stage by hypochlorite treatment. Animals were plated onto 10-cm 8P/NA22 plates for 1 day at 20° until they reached the L2-L3 stage. Worms were then washed off the plate and incubated with 2, 5, or 10 mM ascorbic acid ± 10, 25, or 50 mM 6-OHDA, respectively, for 1 hr on a covered Nutator. Worms were then pelleted and replated onto 8P/NA22 plates without removal of the 6-OHDA and incubated for 3 days at 20° until the worms reached adulthood. Degeneration of CEP neuron dendrites was scored blind on a four-point scale (where 4 = all dendrites intact and 0 = complete loss of dendrites). For scoring, 100 to 200 worms were placed on a freshly prepared 2% agarose pad and immobilized using 225 mM 2,3 butanedione monoxime (Sigma-Aldrich) and 2.5 mM levamisole (Sigma-Aldrich) in 10 mM Na HEPES. Fifty worms were scored per slide in triplicate for each strain assayed.

## Results

### Forward genetic screen for reserpine-sensitive Swip phenotype

An artificial increase in extrasynaptic DA produced by the incubation of nematodes with exogenous DA leads to increased activation of inhibitory DA receptors expressed on cholinergic motor neurons (Chase *et al.* 2004), decreasing the release of acetylcholine from these neurons (Allen *et al.* 2011), resulting in paralysis. Our previous studies demonstrated that *dat-1(ok157)* worms display a paralytic phenotype in water in the absence of exogenous DA that we named Swimming Induced Paralysis, or Swip (McDonald *et al.* 2007). The Swip phenotype exhibited by *dat-1* animals is absent in *cat-2*/TH;*dat-1* double mutants and can be rescued by pretreatment of animals with the *cat-1*/vMAT inhibitor reserpine, a reagent that depletes vesicular DA stores (McDonald *et al.* 2007). Importantly, *dat-1*;*dop-3* animals (Sugiura *et al.* 2005) also lack Swip behavior, establishing Swip as a phenotype that can derive from hyperdopaminergic signaling.

**Figure 1:**
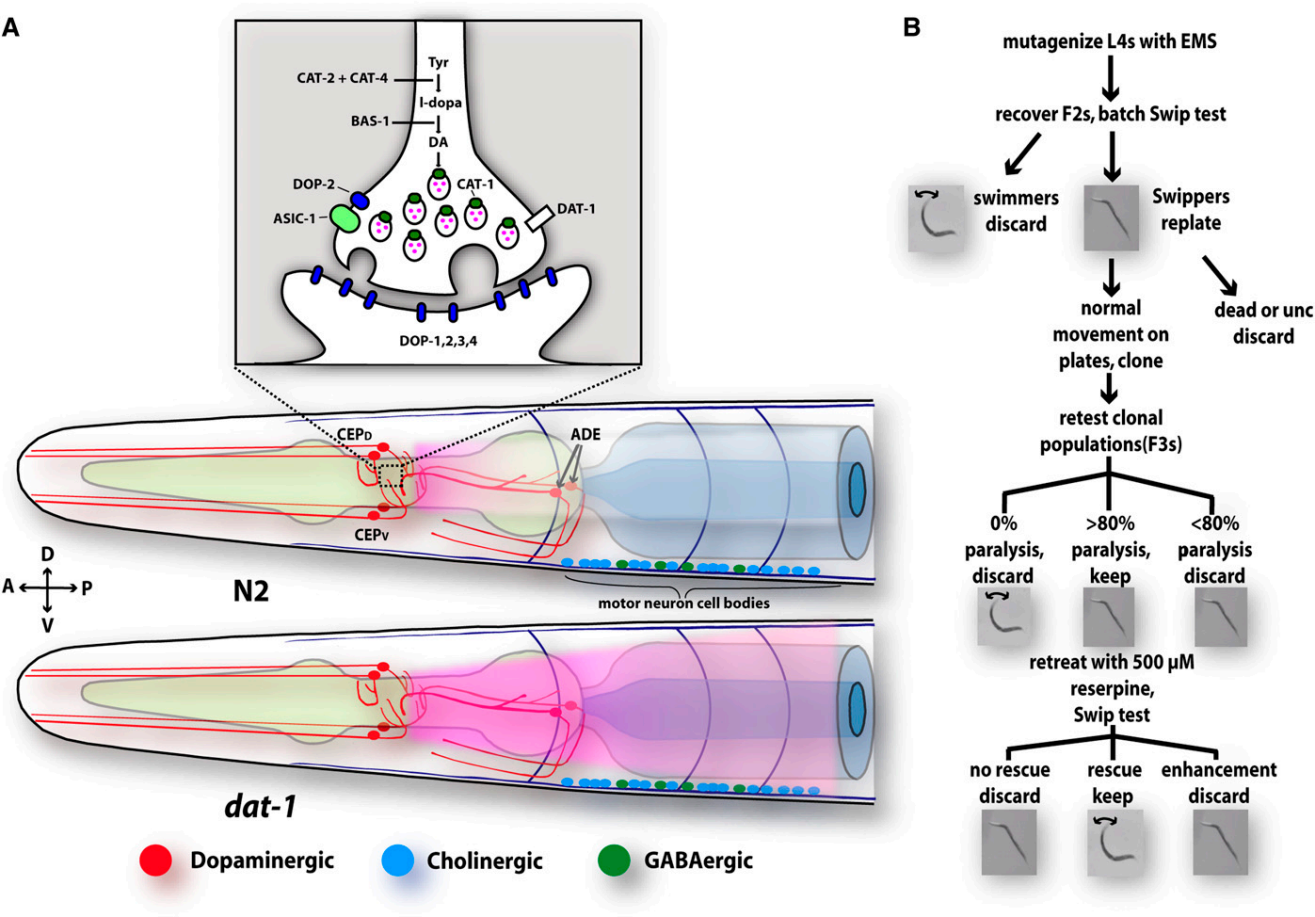
Generation of reserpine-sensitive Swip establishes a phenotype for forward genetic identification of modulators limiting hyperdopaminergic signaling. (A) *C. elegans* hermaphrodites contain eight DA neurons: four CEPs, two ADEs, and two PDEs (not shown). These neurons release DA that can act humorally on the motor circuit through stimulation of the DA receptors DOP-1 and DOP-3 that are coexpressed in cholinergic motor neurons. [Inset: illustration of presynaptic DA terminals with gene products responsible for DA synthesis (CAT-2, CAT-4, BAS-1), packaging (CAT-1), release (DOP-2, acid-sensing ion channel 1; ASIC-1) and inactivation (DAT-1)]. In DAT-1−deficient animals, the release of endogenous DA leads to a much larger increase in extrasynaptic DA, leading to hyperactivation of DOP-3 DA receptors and paralysis. (B) Design of Swip-based forward genetic screen. We synchronized L4 animals with EMS and allowed them to self-fertilize and isolate F1s. F2s were Swip tested in batch, and paralyzing animals were cloned. Animals that did not display grossly normal movement on standard solid medium were eliminated, and F3s were generated and then retested to insure transmission of Swip. Clones that demonstrated at least 80% paralysis (our pilot efforts used >50% but yielded poor stable transmission) were grown on 0.6 mM reserpine to deplete vesicular DA stores and tested for rescue of Swip. Clones that were rescued by reserpine pretreatment were given *vt* mutation designations and their progeny were used in all subsequent experiments.

As schematized in Figure 1A, we hypothesized that incubation of worms in water (but not in isotonic medium; J. A. Hardaway and R. D. Blakely, personal communication) evokes DA release that in wild-type animals is limited in action by DAT-1. In *dat-1* animals, DA cannot be recaptured and spills over to extrasynaptic sites, where it can activate DOP-3 on motor neurons, decreasing cholinergic, neuromuscular signaling (Allen *et al.* 2011), producing Swip.

Because the Swip assay is a simple, rapid, and highly reproducible method for detecting genetic contributions to endogenous DA signaling, we implemented Swip in a forward genetic screen by using multiple, secondary tests to eliminate nonspecific mutants. As outlined in Figure 1B, L4 BY200 (P*_dat-1_*::GFP) animals were mutagenized with EMS, and their F2 progeny batch were tested for Swip. Animals exhibiting Swip were individually cloned, and their progeny (F3s) were tested to determine Swip penetrance, scored as the percent of animals of a clonal population exhibiting Swip. Animals that failed to move normally on plates, and thus where Swip might reflect mutation of genes required for movement more generally (*e.g.*, *unc* mutations), were eliminated. As a tertiary screen, we tested animals for Swip reversal after incubation with reserpine (McDonald *et al.* 2007). Because Swip in the *dat-1* strain is fully penetrant, lines that exhibited ≥80% paralysis were pretreated with 0.6 mM reserpine, and animals were retested for Swip. From this tertiary analysis, we identified mutants that were either unaffected, rescued or, surprisingly, enhanced by reserpine treatment. To date we have sampled ~10,000 mutant haploid genomes, and have isolated 25 primary mutant lines (*vt20-vt44)* that demonstrate a stable Swip phenotype and can be rescued by reserpine pretreatment. Of these 25, we found that 12 lines maintain their phenotype after 3X outcrossing, of which 10 lines are consistently rescued by reserpine pretreatment (Figure 3A). Here we report our genetic, biochemical, and behavioral analysis on four of these lines: *vt21*, *vt22*, *vt25*, and *vt29*.

To demonstrate that *vt21*, *vt22*, *vt25*, and *vt29* possess normal DA neuron morphology, we examined their CEP, ADE and PDE neurons via expression of a transcriptional reporter (supporting information, Figure S1) and found that cell bodies and processes appear unaltered relative to N2 animals. These findings are consistent with our hypothesis that Swip is a hyperdopaminergic phenotype that is unlikely to derive from gross alterations in DA neuron circuitry.

### Isolation of novel *dat-1*−null mutations

If our Swip screen is both specific and robust with respect to recovery of genes that control DA signaling, it should recover animals with *dat-1* alleles, as well as novel mutants. To assess this issue, we first crossed *vt21*, *vt22*, *vt25*, and *vt29* with *dat-1(ok157)*, performing Swip complementation tests on their F1 progeny. From these experiments we found that N2 can complement *dat-1* and each of the mutant strains (Figure S2A) and that *vt25* and *vt29* both complement *dat-1* (Figure S2B) and one another (data not shown), indicating that *vt25* and *vt29* likely do not derive from mutations in *dat-1*. In contrast, *vt21* and *vt22* failed to fully complement Swip when crossed to *dat-1(ok157)* (Figure S2B). Likewise, when we crossed *vt21* and *vt22*, we observed a failure to complement (data not shown), indicating that they are likely alleles of the same gene, that most likely is *dat-1*.

**Figure 2:**
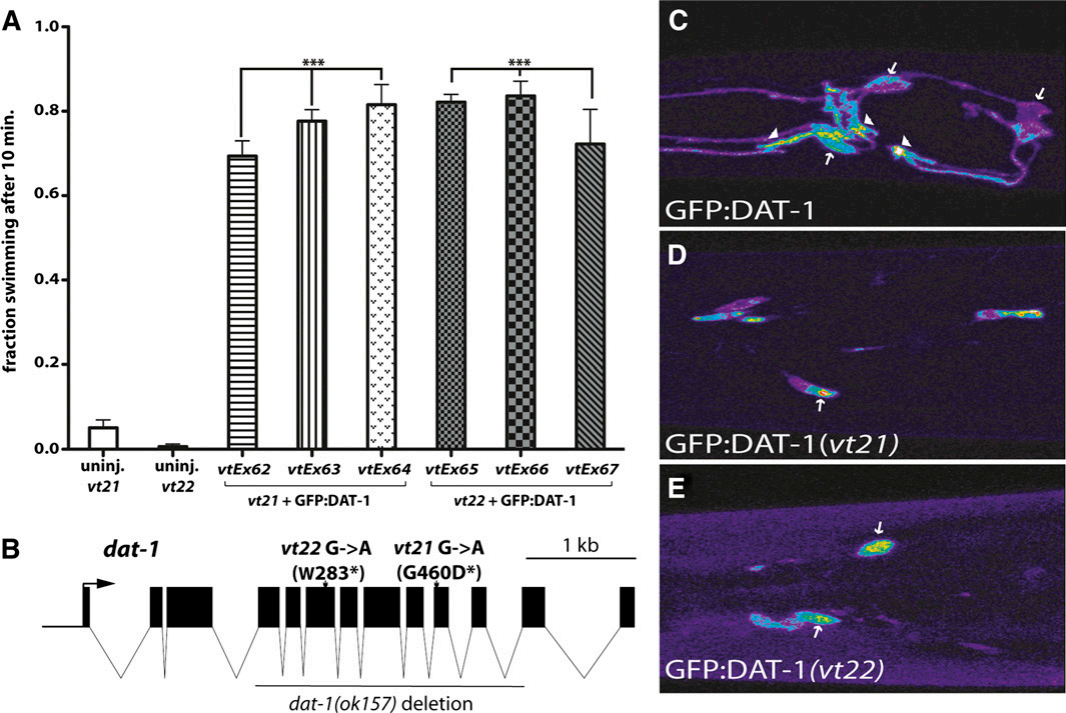
Isolation of novel *dat-1* mutants. (A) GFP:DAT-1 rescues swimming behavior of *vt21* and *vt22* animals. *vt21;lin-15(n765ts)* and *vt22;lin-15(n765ts)* were coinjected with GFP:DAT-1(pRB491) and lin-15(pJM23), and separate transgenic lines were manually scored for paralysis in water. Data are derived from ≥10 trials for each line. Data were analyzed using one-way analysis of variance (ANOVA) with selected Bonferroni post tests; ^***^P < 0.001. (B) Gene structure of *dat-1* and position of mutant nucleotides in *vt21* and *vt22* as verified by DNA sequencing. Also illustrated is the *dat-1(ok157)* allele that derives from a 1.8-kb deletion encompassing exons 4-11 of *dat-1*. In *vt22*, a G→A transition at nucleotide 1687(from ATG start) results in the conversion of tryptophan (T) to a premature stop codon (^*^). In *vt21*, a G→A transition at nucleotide 2395 results in the conversion of a highly conserved glycine (G) to aspartic acid (D). (C−E) Expression and trafficking deficits of *vt21* and *vt22* alleles of *dat-1*. Animals injected with p*_dat-1_*::GFP:DAT-1 (C) display readily detectible expression and localization to CEP and ADE soma (white arrows) and terminals (white triangles) whereas mutant transporters (D and E) are much more weakly expressed and restricted in localization to DA neuron cell bodies. L4 animals were anesthetized and Z-stacks of CEP/ADE neurons were acquired and flattened to generate images of GFP:DAT-1 expression *in vivo*. Each image is representative of three separate transgenic lines that produced equivalent results.

To test this hypothesis, we overexpressed GFP-tagged DAT-1(P*_dat-1_*::GFP:DAT-1) in *vt21* and *vt22* and tested for Swip in lines verified to express GFP in DA neurons. As previously demonstrated for the DAT-1 deletion allele *dat-1(ok157)* (McDonald *et al.* 2007), transgenic expression of GFP:DAT-1 rescued the Swip phenotypes of *vt21* and *vt22* (Figure 2A), suggesting that these mutants reduce DAT activity. Whereas sequencing of the genomic DAT-1 locus in *vt25* and *vt29* yielded a sequence identical to that found in N2 animals, *vt21* and *vt22* were found to bear single base-pair substitutions that were predicted to impact the DAT-1 coding sequence (Figure 2B). The DAT-1 gene contains 13 exons that encode a 615 amino-acid protein with 12 transmembrane domains and intracellular N and C-termini (Jayanthi *et al.* 1998). *vt21* bears a G→A substitution at the beginning of exon 10 that results in the conversion of a highly conserved glycine residue to aspartic acid (G460D). G460 (conserved in hDAT) is predicted to lie in the small extracellular hairpin turn between transmembranes 9 and 10 (Figure S3). *Vt22* possesses a G→A substitution in the middle of exon 6 that results in a stop codon at amino acid 283, as opposed to a tryptophan (W283^*^).

To examine the deleterious impact of the DAT-1 mutations identified in *vt21* and *vt22* lines, we implemented a previously described heterologous expression protocol (Nass *et al.* 2005). Our assay of [^3^H]DA transport activity in COS-7 cells revealed that both *dat-1(vt21)* and *dat-1(vt22)* display significantly reduced DA uptake relative to that displayed by WT HA:DAT-1 (Figure S3C). Whereas *dat-1(vt22)* produced no significant [^3^H]DA uptake above nontransfected cells, *dat-1(vt21)* exhibited low, but detectible, DA transport activity (Figure S3C). Analysis of total and cell-surface protein extracts demonstrated that *dat-1(vt21)* and *dat-1(vt22)* exhibit reduced cell-surface expression levels and a premature truncation product respectively relative to WT DAT-1(Figure S3D). Furthermore, whereas GFP:DAT-1 expression rescued the Swip phenotype of *dat-1(*ok157) (Figure S3E), neither GFP:DAT-1(G460D) nor GFP:DAT-1(W283stop) rescued Swip. Confocal analysis of wild-type GFP:DAT-1 expression in CEP neurons revealed moderate levels of transporter expression in DA cell bodies (Figure 2C, white arrows), low levels in dendrites and axons, and high expression in presynaptic terminals (white arrowheads). In contrast, GFP:DAT-1(G460D) demonstrated robust CEP cell body expression but failed to label CEP processes and terminals (Figure 2D). Animals expressing GFP:DAT-1(W283stop) demonstrated lower levels of GFP signal than was observed in GFP:DAT-1 or GFP:DAT-1(G460D), with all labeling confined to CEP cell bodies (Figure 2E). Together, these findings provide *in vitro* and *in vivo* support for the determinants of Swip in the *vt21* and *vt22* lines as derived from *dat-1* alleles. Because we did not observe mutations in DAT-1 in *vt25* and *vt29*, we further mapped the sites of these mutations as described in *Materials and Methods*. We found that *vt25* maps to LGIII, whereas *vt29* maps to LGX (Figure S4). No genes known to impact DA signaling lie in the region mapped in *vt25*. The *vt29* region harbors the *dop-4* gene. Sequencing of this gene in *vt29* animals revealed no mutations, indicating that the altered DA signaling of *vt29* is produced through a novel mechanism. These findings confirm that *vt25* and *vt29* do not harbor Swip-causing *dat-1* alleles, nor do they involve mutations in the same gene.

### *vt25* and *vt29* exhibit *dat-1*−like locomotive behaviors but distinct sensitivities to exogenous DA

**Figure 3:**
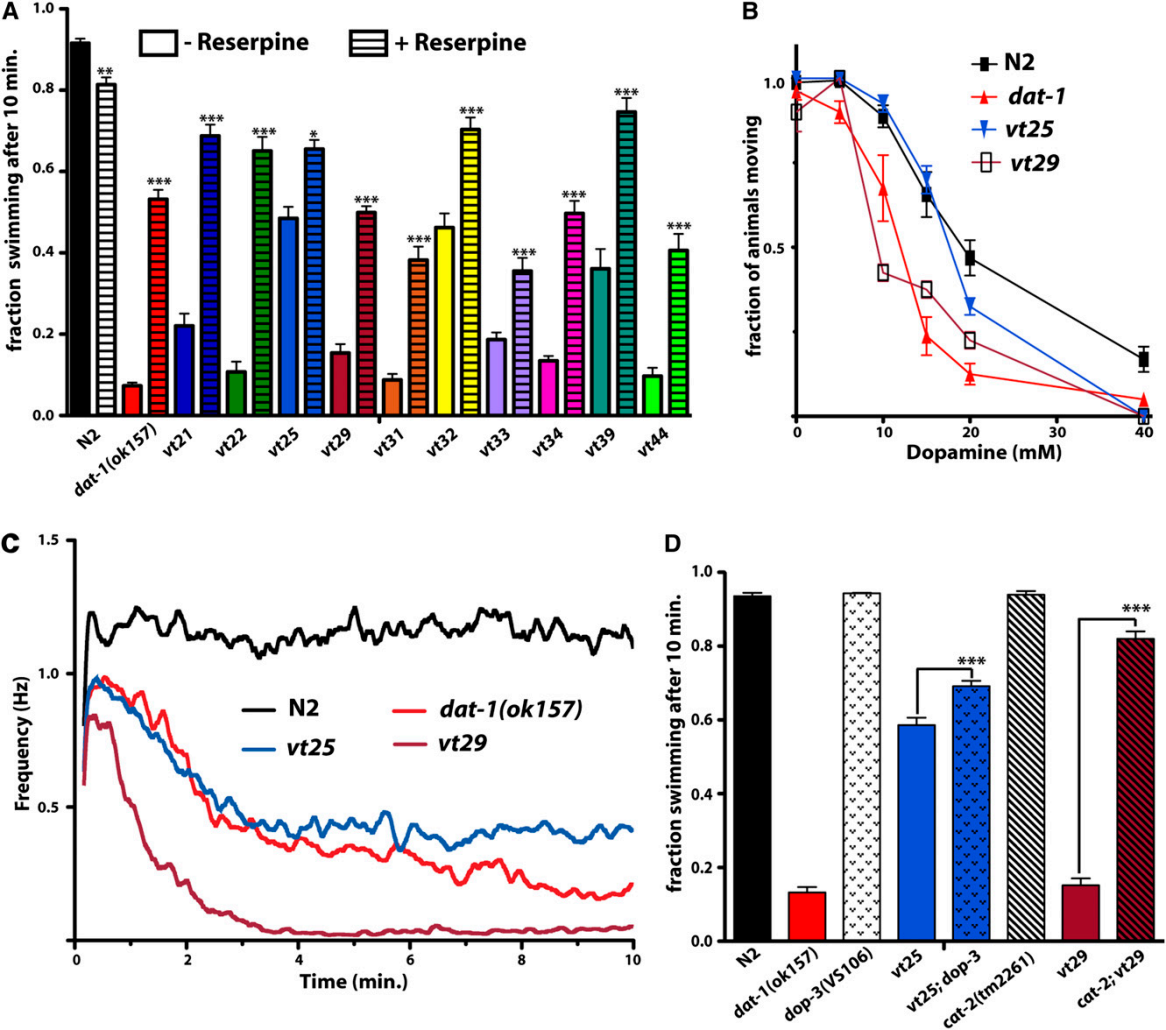
*vt25* and *vt29* demonstrate alterations in DA-dependent locomotory behaviors. (A) After a screen of ~10,000 haploid genomes, 25 *vt* mutant lines were outcrossed, of which 10 were found to maintain their reserpine sensitivity. Populations of at least 10 worms were assayed in a single well and after 10 min, with animals scored as no. animals swimming/total animals. Each bar represents at least 20 assays performed over several experimental days by multiple, blinded experimenters. Drug treatment effects were analyzed using an unpaired Student's *t*-test comparing against basal Swip activity for that line, where ^*^P < 0.05, ^**^P < 0.01, and ^***^P < 0.001 and error bars represent SEM. (B) *Dat-1* and *vt29* displayed enhanced sensitivity to exogenous DA on a solid substrate, whereas *vt25* maintained an N2-like DA dose response. For these assays, 10 L4 stage worms were placed on plates containing increasing concentrations of exogenous DA, incubated for 20 min, and then scored for 10 sec as paralyzed or moving. Dose−response curves were compared using two-way ANOVA with Bonferroni posttests comparing mutants to N2, where *dat-1* + *vt29* were all found to be significantly different from N2 with a *P* < 0.001 at 15 + 20 mM DA. Data derive from at least eight tests per strain per concentration. Error bars represent SEM. (C) *vt25* demonstrates a greater average frequency of swimming at later time points as compared with *dat-1* animals, whereas *vt29* paralyzes faster. Individual animals were recorded using a video capture system and then analyzed with custom-designed Thrasher software that assigns multiple linear elements projecting from the worm centroid. The position of these linear elements are tracked and converted off-line to movement frequency as a function of time. Batch conversions are generated, providing mean values and SEM along moving averages. Error bars are not shown in these plots for simplicity. Average thrashing plots were analyzed using two-way ANOVA with Bonferroni's multiple comparison analysis. *vt25* and *vt29* swimming frequencies were found to be significantly reduced from N2 after 58 sec and 39 sec, respectively. Although we observed no overall significant difference in these assays in comparing *dat-1(ok157)* and *vt25*, the thrashing frequency of *vt29* was found to be significantly reduced from *dat-1(ok157)* between 52 sec and 2 min 25 sec. (D) *Swip* behavior of *vt25* and *vt29* is dependent on DOP-3 and CAT-2, respectively. The figure represents the mean swimming behavior as measured by manual scoring. Data derive from observations from >24 trials for each strain. For *vt25*; *dop-3(vs106)* and *cat-2(tm2261)*; *vt29*, the *vt* genotypes were confirmed via an independent complementation test. Data were analyzed using one-way ANOVA with multiple Bonferroni posttests where ^***^P < 0.001 and error bars represent SEM.

To determine the similarity of *vt25* and *vt29* behavior to that of *dat-1*, we conducted a series of DA signaling−dependent locomotor tests. We also assayed *dat-1(vt21)* and *dat-1(vt22)* in parallel to explore whether this test might reveal *dat-1* allele-specific effects. Using an automated assay of swimming behavior (Matthies *et al.* 2006; McDonald *et al.* 2007), we found that *vt25* lacked a fully penetrant Swip phenotype, as evident by its ~50% paralysis in manual scoring (Figure 3A) and an average ~0.5 Hz thrashing frequency by our final assay time point (10 min) under automated recording (Figure 3C). In contrast, *vt29* displayed fully penetrant Swip, yielding ~100% paralysis in manual scoring assays (Figure 3A), and an even more rapid rate of paralysis than *dat-1* (Figure 3C and Table 1). In these and further assays, we determined that *dat-1(vt21)* and *dat-1(vt22)* behave similar to the *dat-1(ok157)* strain (Figure S5 and Table 1).

**Table 1:** Kinetic attributes of paralysis and reversion

	Maximal Frequency, Hz	Latency to Paralyze, sec	Reversion Incidence*^a^*	Reversion Frequency, Events/Animal	Reversion Probability*^b^*	Time to First Reversion, sec	Average Reversion Event Length, sec
*N2* (n = 52)	1.76 ± 0.01	N/A	N/A	N/A	N/A	N/A	N/A
*dat-1(ok157)* (48)	1.45 ± 0.04*^c^* ^*^	174 ± 17.5	0.25	3.22 ± 0.571	0.031 ± 0.006	145 ± 35.8	5.77 ± 1.4
*dat-1(vt21)* (43)	1.28 ± 0.04^*^	130 ± 14.4	0.365	1.76 ± 0.302	0.015 ± 0.003	185 ± 32.3	4.02 ± 0.93
*dat-1(vt22) (*47)	1.24 ± 0.04^***^	131 ± 14.5	0.391	2.00 ± 0.271	0.020 ± 0.004	112 ± 17.7	4.54 ± 0.93
*vt25* (47)	1.28 ± 0.04^*^	178 ± 21.8	0.267	3.50 ± 0.620	0.032 ± 0.01	183 ± 67.1	4.22 ± 0.88
*vt29* (37)	1.13 ± 0.04^***^	81.2 ± 6.05^***^	0.0540*^d^* ^*^	2.50 ± 0.500	0.018 ± 0.007	146 ± 108	3.59 ± 0.69
*vtIs18* (42)	1.64 ± 0.02*^c^*^ns^	N/A	N/A	N/A	N/A	N/A	N/A
*vt25*; *vtIs18* (38)	1.45 ± 0.03	337 ± 61.9^*^	0.333	N/A	N/A	N/A	N/A
*vt29*; *vtIs18* (44)	1.45 ± 0.02	165 ± 22.4	0.294	3.90 ± 0.706	0.051 ± 0.019	107 ± 30.7	4.43 ± 1.14
M9	
N2 (53)	1.76 ± 0.01	N/A	N/A	N/A	N/A	N/A	N/A
*dat-1(ok157)* (56)	1.66 ± 0.02	166. ± 34.1	0.810*^d^* ###	5.06 ± 0.774	0.052 ± 0.014	110 ± 11.7	4.18 ± 1.10
*vt29* (53)	1.29 ± 0.03^****^	298 ± 24.5	0.640*^d^* ##	4.19 ± 0.647	0.040 ± 0.008	171 ± 25.7	4.20 ± 0.76

Asterisks indicate a significant difference to *dat-1(ok157)*, as determined by one-way analysis of variance with Bonferroni's multiple comparision test, where ^*^P < 0.05, ^**^P < 0.01, and ^***^P < 0.001. Numeral signs (#) indicate a comparison within the same genotype between water and M9.

a Reversioni = fraction of animals that reverse from paralysis.

b Reversion probability = total time spent in reversion/total time after paralysis onset.

c Comparison with N2(ns = not significant).

d χ^2^ test.

The Swip of *dat-1* animals requires the function of the Gα_o_-coupled DA receptor DOP-3 (Chase *et al.* 2004; McDonald *et al.* 2007; Allen *et al.* 2011) that is expressed by body wall muscle and ventral cord motor neurons. As with *dat-1(ok157)* animals, *dat-1(vt21)* and *dat-1(vt22)* paralysis was completely suppressed in a cross to *dop-3* (Figure S5). *Vt25* paralysis was modestly but significantly rescued by *dop-3* (Figure 3D). We could not examine rescue of *vt29* with *dop-3* because they both map to the same chromosome. Therefore, we crossed this line to a line deficient in the rate-limiting enzyme in DA biosynthesis, tyrosine hydroxylase *(cat-2*), as *cat-2* suppresses the Swip of *dat-1(ok157)* (McDonald *et al.* 2007). As with *dat-1* alleles, we found that *cat-2* completely suppressed the paralysis behavior of *vt29* (Figure 3D).

The application of exogenous DA to worms on solid substrate induces motor slowing and paralysis (Schafer and Kenyon 1995). We found that *dat-1* animals transferred to plates containing increasing amounts of DA displayed a twofold increase in DA sensitivity when compared with N2 animals (Figure 3B and Figure S5), possibly reflecting a lack of DAT-1−dependent clearance of exogenous DA once the catecholamine permeates the cuticle. *Vt29* displayed sensitivity to exogenous DA like that of *dat-1* (Figure 3B). In contrast, *vt25* exhibited a DA dose-response more similar to N2 (Figure 3B).

### Automated analysis of Swip behavior reveals a differential impact on thrashing behavior for *vt25* and *vt29*

**Figure 4:**
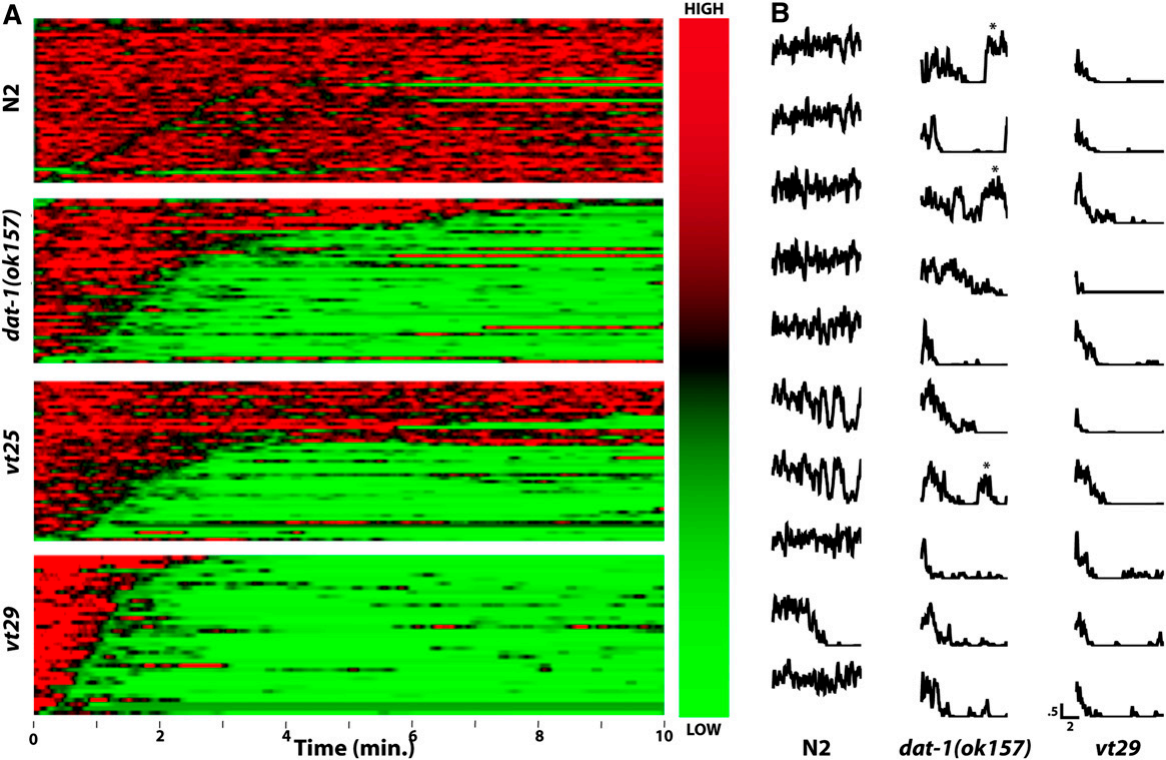
*vt25* and *vt29* display distinct paralytic attributes. (A) Heat map analysis for N2, *dat-1(ok157)*, *vt25*, and *vt29* animals, generated using SwimR software as described in *Materials and Methods*. Individual swimming frequencies as a function of time, color coded as shown by the vertical bar on the right, are stacked with the fastest animals to initiate paralysis for each genotype placed on the bottom. (B) Individual traces of N2, *dat-1* and *vt29* behavior illustrate that a proportion of *dat-1* animals demonstrate spontaneous reversal of paralysis (reversals marked with ^*^), behavior that is rarely seen with *vt29* animals. Traces shown reflect individual traces from the full data set used for heat map generation, selected using a random number generator. Full quantitative analysis of reversal behavior elements is provided in Table 1. Scale bar for frequency and time in min is shown below *vt29* plots.

To more precisely quantify the thrashing behavior of our *vt* mutants, we developed software tools (SwimR; see *Materials and Methods*, J. A. Hardaway and J. Wang, unpublished data) that can provide for a more detailed kinetic analysis of individual animals that may reveal patterns of behavior not readily detected in population averages (Table 1). For visualization, our software plots each animal's swimming behavior horizontally (Figure 4A), assigning a color code that ranges from red (high frequency) to green (low frequency) for each time block and that orders the animals within each genotype so that more rapid paralyzers are displayed at the bottom and slower paralyzers (or relatively constant swimmers) are displayed at the top. These analyses revealed several significant differences between the mutants. All mutants recovered in the screen demonstrated significantly reduced maximal thrashing frequencies in water, with *vt29* being the most impacted, and accompanied by a greater percentage of animals displaying lower thrashing frequency values (Figure S6). With respect to paralysis, *vt25* animals again resembled *dat-1*, whereas *vt29* differed from these lines with a significantly reduced latency to paralyze. Although by definition, all lines paralyze in water, our single-worm analyses revealed that *dat-1* and *vt25* lines feature a significant number of animals that revert back to relatively normal swimming behavior that we tabulated as reversion incidence (no. revertants/no. paralyzers)) and reversion probability (time in reversion/time after paralysis). *Vt29* demonstrated a significantly reduced reversion incidence from other lines (and *dat-1*), which can be readily detected in both the heat map plots and in randomly selected individual thrashing traces (Figure 4B). When animals that did revert were analyzed separately, we found that the reversion frequency (no. events/animal), time to first reversion, and the average reversion event length did not differ among all mutant strains (Table 1). These analyses suggest that differences exist in the functional impact of *dat-1*, *vt25*, and *vt29* mutations, prompting further investigation of the differential behavior of these lines.

### Evaluation of *vt25* and *vt29* sensitivity to osmolarity

**Figure 5:**
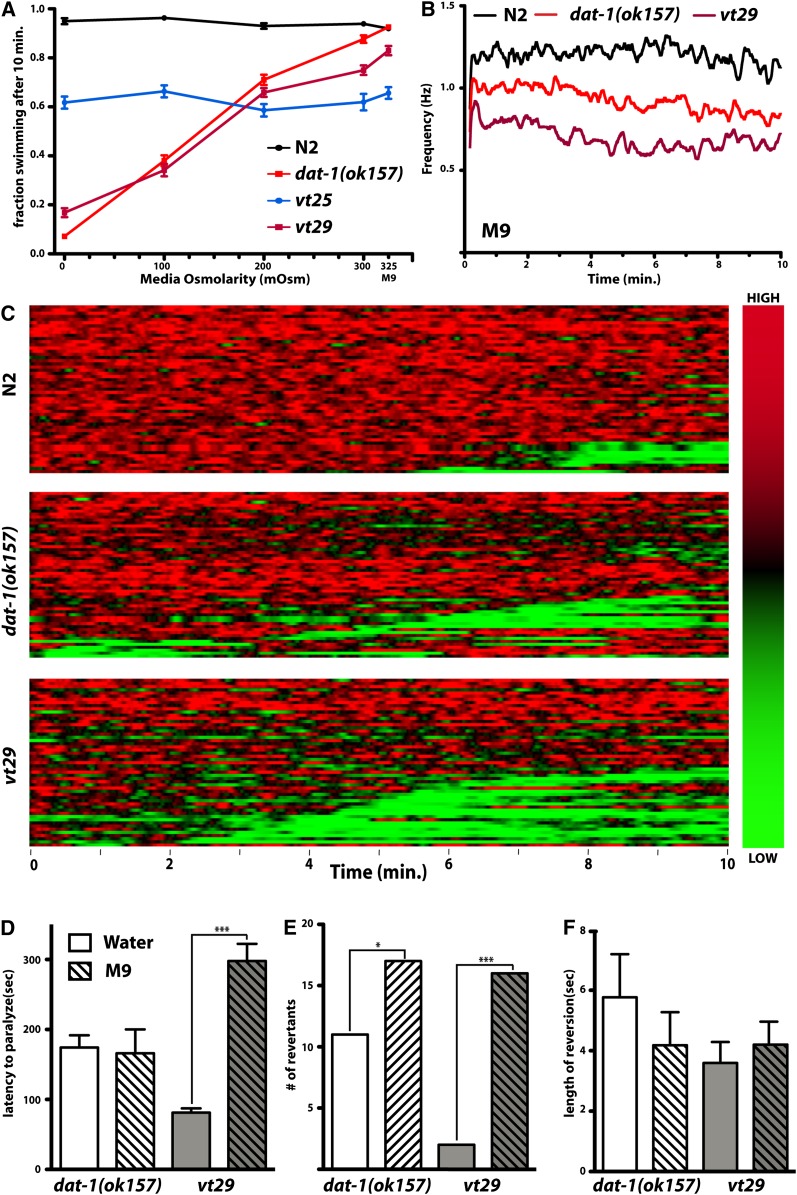
Swip in *dat-1*, *vt25* and *vt29* are variably suppressed at high buffer osmolarities. (A) The Swip penetrance of *dat-1* and *vt29* is reduced by increasing the osmolarity of the swimming medium, whereas *vt25* displays no sensitivity to changes in osmolarity. Manual Swip assays were performed as described previously with water supplemented with sucrose to produce 100, 200 + 300 mOsm. M9 was used as a high osmolarity control and was experimentally determined to be ~325 mOsm. Data were analyzed using two-way ANOVA, with the goal of evaluating a genotype X molarity interaction. Significant interactions were found for *dat-1* (*P* < 0.0001) and *vt29* (*P* < 0.0001)), whereas *vt25* displayed a nonsignificant interaction (*P* = 0.2385). Bonferroni *post hoc* comparisons of each genotype at each osmolarity were performed to assess the ability of each buffer to suppress Swip. (B) *vt29* bears an enhanced Swip penetrance in M9 as measured via automated thrashing analysis. Individual traces of N2, *dat-1*, and *vt29* were acquired as described previously except M9 was used in lieu of water. Data were analyzed using two-way ANOVA with Bonferroni posttests along the running averages. The thrashing frequency of *vt29* was significantly reduced from *dat-1* from 3 min 14 sec to 3 min 17 sec and 4 min 37 sec to 4 min 39 sec and *dat-1* significantly reduced from N2 from 5 min 42 sec to 5 min 45 sec and 8 min 50 sec to 8 min 52 sec. C. Heat map analysis using SwimR software for N2, *dat-1(ok157)* and *vt29* animals in M9 generated as described in *Materials and Methods*. (D) Quantitative analysis of Swip behavior displayed graphically in C. M9 buffer significantly increases the latency to paralyze for *vt29*, but not for *dat-1*. Mean latency values were compared using an unpaired, two tailed *t*-test (*P* < 0.001). (E) M9 buffer significantly increases the reversion incidence of both *dat-1* and *vt29*. The distribution of revertants between the two buffers was compared using a χ^2^ test. (F) M9 buffer does not alter the average length of reversion events in either *dat-1* or *vt29*. See Table 1 for complete analysis.

Previously, we demonstrated that the *dat-1* Swip phenotype is highly dependent on the osmolarity of the swimming medium (J. A. Hardaway, personal communication to Worm Breeder's Gazette), with a near-complete loss of Swip when these animals are subjected to aqueous solutions buffered to 300 mOsm with sucrose, or in M9 (325 mOsm). We used manual scoring of the fraction of animals paralyzed at 10 min to assay swimming behavior of N2, *dat-1*, *vt25*, and *vt29* as a function of medium osmolarity. N2 animals, as expected, maintained a relatively constant swimming rate regardless of osmolarity (Figure 5A), whereas *dat-1* animals displayed the expected loss of paralysis with increasing osmolarity. *Vt29* animals exhibited a virtually identical sensitivity to osmolarity as *dat-1*, except a slight, yet significantly increased paralysis at greater osmolarities. Remarkably, *vt25* animals displayed a lack of sensitivity to the osmolarity of solutions used in swimming assays. Although only minimal paralysis of *dat-1* and *vt29* animals was detected in M9 medium using manual scoring, automated analyses revealed that both *dat-1* and *vt29* exhibited reduced average rates of movement (Figure 5B). At the single-worm level, several parameters distinguished *dat-1* and *vt29* in M9 medium (Figure 5, C−F; Table 1). V*t29* animals could not achieve the same maximal swimming frequency as *dat-1* animals in M9, and unlike water, *dat-1* maximal rates were not significantly different from N2 (Table 1). In M9, *vt29* animals exhibited a significantly greater latency to paralyze than *dat-1* animals (Table 1 and Figure 5D), opposite to their relative sensitivities in water. Both *dat-1* and *vt29* lines demonstrated an increase in the number of revertants in M9 as compared with water (Figure 5E) without changing the length of reversion events (Figure 5F), although this effect was greatest in *vt29* animals. Therefore, *vt29* are more likely to paralyze in M9 than *dat-1* but as a population reach paralysis more slowly and are more likely to reverse from paralysis than when assayed in water.

### Suppression of Swip in *vt25* and *vt29* with DAT overexpression

**Figure 6:**
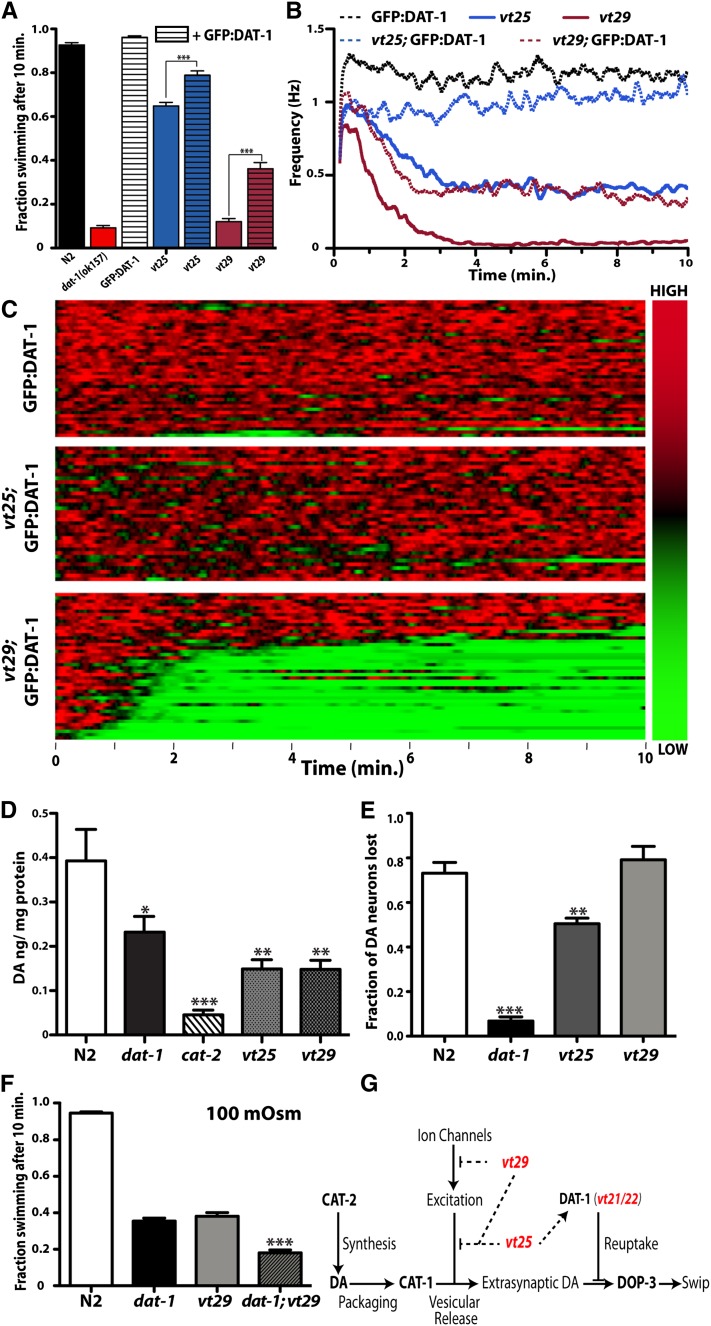
Behavioral analysis of GFP-DAT overexpression, biochemical analysis, and neurotoxic profiles of *vt25* and *vt29*. (A) p*_dat-1_*::GFP:DAT-1 transgene suppresses Swip behavior of *vt25* and *vt29* lines. A line containing an integrated functional p_dat-1_:: GFP-DAT-1 (*vtIs18*) transgene was crossed onto *vt25* or *vt29*. Lines were tested manually over at least three experimental days with >72 trials tested for each strain. Data were analyzed using a one-way ANOVA with multiple Bonferroni posttests. ^***^P < 0.001. (B) Automated thrashing analysis of *vt25* and *vt29*, generated as previously described, demonstrate rescue of Swip in cross to *p_dat-1_*: GFP-DAT-1 (*vtIs18*). (C) Heat map representations of *vt25*; *vtIs18* demonstrate that whereas the overexpression of GFP:DAT-1 fully rescues the paralysis of *vt25* (compare with Figure 4A), with transient bouts of paralysis evident, whereas *vt29*; *vtIs18* animals demonstrate a reduction in Swip penetrance relative to *vt29* (compare with Figure 4A) and are not fully rescued. (D) Analysis of DA levels in *vt25* and *vt29*. *dat-1(ok157)*, *cat-2(tm2261)*, *vt25*, and *vt29* lines all contain significantly reduced DA levels as compared with N2. For each strain, DA was extracted from synchronized L4 populations and measured using HPLC as described in *Materials and Methods*. Data were analyzed using a one-way ANOVA with Dunnett's multiple comparison test. ^*^, ^**^, ^***^ indicate a significant reduction from N2 where *P* < 0.05, 0.01, and 0.001 respectively. Error bars represent SEM and derive from >7 experiments for each strain. (E) DA neurons in N2 are highly sensitive to treatment with the neurotoxin 6-OHDA, which requires the presence of DAT-1. *vt29* is fully sensitive to 6-OHDA treatment, whereas *vt25* is partially resistant. Treatments were performed as described in *Materials and Methods*. Data were analyzed using a one-way ANOVA with multiple Bonferroni posttests where ^**^ and ^***^ indicate a significant reduction from N2 where *P* < 0.01 and *P* < 0.001, respectively. (F) *dat-1(ok157)*; *vt29* demonstrates an enhanced Swip penetrance relative to *dat-1(ok157)* and *vt29* alone at an intermediate osmolarity (100 mOsm). Lines were tested manually over at least three experimental days with >48 trials for each strain. Data were analyzed using one-way ANOVA with multiple Bonferonni posttests. *dat-1(ok157)*; *vt29* was found to be significantly reduced from either mutant line alone. (G) Model for differential contributions of *swip-6(vt25)* and *swip-10(vt29)* to Swip. *Vt21/vt22* impacts DAT activity directly through mutation of DAT-1 and loss of function. *swip-10* is hypothesized to function in a pathway parallel and upstream of DAT-1 activity, whereas *swip-6* acts to constrain both extracellular DA release and reuptake.

Although we did not detect mutations at the DAT-1 locus in *vt25* and *vt29*, these mutations may still impact DA clearance, either through a functional impact on DAT-1 at DA terminals or through changes in the somatic export of DAT-1 protein. To assess this issue, we crossed *vt25* and *vt29* onto a line containing an integrated p*_dat-1_*::GFP:DAT-1 transgene and monitored swimming behavior. Overexpression of GFP:DAT-1 restored swimming behavior of *vt25* (Figure 6A) to near wild-type levels. Single-worm analyses (Figure 6, B and C) demonstrated that although Swip is largely rescued when overexpressing DAT on the *vt25* background, short paralytic bouts are still evident. GFP:DAT-1 overexpression only modestly suppressed *vt29*. Automated analyses revealed that the partial suppression of *vt29* by overexpression of GFP:DAT-1 does not derive from an increased reversion probability or latency to paralyze (Table 1) but rather appears to result from a reduction in Swip penetrance, with a reduced fraction of animals swimming at or near the rates observed with *vt29* alone (Figure 4A compared with Figure 6C). To examine whether the findings from the rescue experiments with p*_dat-1_*::GFP:DAT-1 reflect a failure to synthesize or traffic the transporter, we examined the impact of *vt25* and *vt29* backgrounds on the localization of GFP:DAT-1 to the soma, dendrites, axons, and presynaptic terminals of CEP, ADE, and PDE neurons. We could detect no changes in the pattern of GFP:DAT-1 localization with the *vt25* and *vt29* strains, comparing animals with GFP:DAT-1 expressed on an otherwise wild-type background to (Figure S7).

### *vt25*, *vt29*, and *dat-1* have reduced tissue DA content

One reason for *vt29* to possess a stronger, DA-dependent Swip phenotype than *dat-1(ok157)* would be for *vt29* to store excess DA in synaptic vesicles that, upon release, could produce greater levels of extrasynaptic DA than seen with wild-type DA levels and a loss of DAT-1−mediated DA clearance. To investigate this issue, we measured DA levels in *vt25* and *vt29* animals in parallel with assays of N2, *dat-1(ok157)*, and *cat-2(tm2261)* lines. As expected, *cat-2(tm2261)* displayed a highly significant reduction in DA levels compared with N2 (Figure 6D). *Dat-1* DA levels were also reduced, in keeping with findings of reduced tissue DA content in DAT knockout mice believed to derive from a need to recycle extracellular DA to maintain vesicular stores (Giros *et al.* 1996). Interestingly, both *vt25* and *vt29* also displayed reduced DA levels, suggesting that, analogous to *dat-1*, *vt25*, and *vt29* animals produce a hyperdopaminergic phenotype that precludes the maintenance of normal presynaptic DA levels.

### *vt25 and vt29* demonstrate differential protection against 6-OHDA neurotoxicity *in vivo*

The reduction in DA levels in *vt25* and *vt29*, in the context of a hyperdopaminergic phenotype, raised the possibility that the mutations harbored by these lines produces a loss of DAT-1 loss function that might be masked by other effects and thus not detected via the methods we had used to this point. Our laboratory previously demonstrated that treatment of worms with 6-OHDA results in a necrotic loss of DA neurons and that this process requires transport of the compound by presynaptic DAT-1 to produce neural degeneration (Nass *et al.* 2002). This phenotype presented us with an opportunity to more directly examine the function of DAT-1 in *vt25* and *vt29* lines *in vivo*. Consistent with our published studies, we observed degeneration of DA neurons in N2, but not *dat-1* animals, after an acute (1 hr) treatment with 6-OHDA (Figure 6E). 6-OHDA was significantly less effective in lesioning DA neurons in *vt25* animals compared with N2, suggesting that the gene responsible for Swip in this line normally plays a role in modulating DAT-1 activity. In contrast, *vt29* demonstrated full sensitivity to the toxin, consistent with the mutation in this line impacting a process parallel to DAT-1 in constraining DA signaling.

### *Dat-1* and *vt29* exhibit additivity for Swip when assayed under hypotonic conditions

With evidence that *vt29* impacts DA signaling in a DAT-1−independent pathway, we returned to our assessment of Swip behavior under hypotonic conditions, examining paralysis at an intermediate osmolarity using single and double mutant lines. At a medium osmolarity of 100 mOsm, we observed a significant increase in Swip in the *dat-1;vt29* double mutant, as compared with the Swip penetrance of *dat-1* or *vt29* alone (Figure 6F) and consistent with the mutation harbored by *vt29* impacting a pathway parallel to that impacted by *dat-1*(Figure 6G).

## Discussion

The *C. elegans* gene T23G5.5 (DAT-1) was the first invertebrate SLC6 family member to be cloned and characterized, revealing structural and functional characteristics similar to those of vertebrate catecholamine transporters (Jayanthi *et al.* 1998). *In vitro* heterologous expression studies revealed DAT-1 to exhibit substrate specificity for DA over other catecholamines and to be antagonized by imipramine, amphetamine, and cocaine. Subsequent *in vivo* efforts (Nass *et al.* 2002) demonstrated that a 700-bp DNA element immediately upstream of the DAT-1 transcription start site confers expression of GFP in all *C. elegans* DA neurons (CEP, ADE, PDE in the hermaphrodite). Nass and coworkers capitalized on the visibility of DA neurons in living nematodes to monitor neurotoxin (6-OHDA)-induced DA neuron degeneration and demonstrate the dependence of cell death on toxin accumulation by DAT-1.

The discovery of a DAT-1 dependent phenotype provided an initial opportunity to implement forward genetic approaches for the identification of 6-OHDA toxicity suppressors (Nass and Blakely 2003; Nass *et al.* 2005). As the basis for this screen derives from sensitivity to an exogenous agent, the utility of the 6-OHDA screen for the elucidation of molecules regulating endogenous, DAT-1−dependent DA synaptic events is limited. Therefore, we sought to identify behavioral alterations arising from compromised, endogenous DA signaling, as revealed in studies of a genetic loss of DAT-1. Although loss of DAT in mice produces a dramatic motor phenotype (Giros *et al.* 1996), and exogenous DA can paralyze worms (Schafer and Kenyon 1995; Sawin *et al.* 2000; Chase *et al.* 2004), deletion of a large portion of the *C. elegans* DAT-1 gene fails to induce an overt motor phenotype on solid substrate. DA neurons in the worm are mechanosensitive (Sawin *et al.* 2000), and thus we reasoned that insufficient physical stimulation is present under normal culture conditions to elicit sufficient DA release, thereby reducing the sensitivity of measures focused on a *dat-1* mutation. Wild-type worms placed in water thrash rapidly for 20 min or longer, and we considered whether this more vigorous mode of motion might trigger more elevated DA release. Indeed, when we placed *dat-1(ok157)* animals in water, we discovered that these animals swim briefly at normal rates and then paralyze—a phenotype we termed Swip (McDonald *et al.* 2007). Swip in *dat-1* animals requires the biosynthesis of endogenous DA, packaging of DA into synaptic vesicles, and activation of the DOP-3/Gα_o_ pathway (McDonald *et al.* 2007). The current study confirms that Swip can be used to screen for molecular alterations that impact DAT-dependent clearance of DA.

In our initial screen, many of the lines we identified, when retested in a subsequent generation, demonstrated incomplete penetrance of the Swip phenotype, and, thus we chose to save only those clones in which at least 80% of animals in subsequent generations exhibited Swip. The current report represents an analysis of ~10,000 haploid genomes, where we uncovered 10 stable lines originating from mutations in at least three distinct genes, as determined through direct sequencing, rescue experiments, mapping, and complementation analyses. Visualization of GFP-labeled DA neurons on the mutant backgrounds verified that gross morphologic deficits of DA neurons do not account for the Swip phenotype of the lines presented in the current report. Our design also reduced the probability that mutations recovered would arise in shared elements of neurotransmitter release machinery or determinants of cholinergic and GABAergic signaling, as these lines would be predicted to exhibit locomotor dysfunction on solid substrate. Evidence that the lines recovered in our screen (1) display *cat-2* and/or *dop-3* reversal; (2) can be rescued by the presynaptically directed vMAT/*cat-1* inhibitor reserpine; (3) include two novel *dat-1* alleles; (4) produce changes in DA levels (for *vt25* and *vt29*); and (5) (for *vt25*) reduce 6-OHDA sensitivity lead us to infer that the approach we have used provides for a preferential identification of presynaptically acting genes. Of course, we cannot rule out that our design and subsequent tests could recover mutants acting postsynaptically, although it seems more likely that a screen to identify suppressors of *dat-1*−mediated Swip would be more useful in recovering such animals (Wani *et al.* 2012). Finally, although we chose not pursue them, we also identified multiple lines that exhibited *enhanced* Swip after reserpine treatment, which could reflect an influence of octopamine, tyramine, or serotonin, as these neurotransmitters can also modulate motor activity and their vesicular packaging by CAT-1 should also be reserpine-sensitive (Duerr *et al.* 1999; Alkema *et al.* 2005).

Two of the lines recovered in our screen harbor point mutations in the coding region of DAT-1. *Vt22* bears a nonsense mutation, predicted to truncate the DAT-1 protein at Trp283, whereas *vt21* possesses a missense mutation predicted to generate a nonconservative substitution of Gly for Asp at amino acid 460. All eukaryotic SLC6 family members possess a Gly residue at this position, although ours is the first study to demonstrate a functional significance of this site *in vivo*. Importantly, our studies of *vt21* and *vt22* provide critical proof of concept that a Swip-based, forward genetic screen can target genes critical for the regulation of DA signaling.

In addition to the isolation of new *dat-1* alleles, we present a functional characterization of two additional stable lines exhibiting Swip, *vt25* and *vt29*. These two lines share reserpine-sensitive Swip with *dat-1(ok157*), *dat-1(vt21)* and *dat-1(vt22)*, and like these mutants, their Swip is suppressed by *dop-3* or *cat-2* mutations. Complementation analyses and mapping of the mutant genes harbored by *vt25* and *vt29* revealed that they bear mutations in two independent loci that do not overlap or contain mutations in known regulators of DA signaling and thus likely represent novel genetic components of DA signaling.

In parallel with *dat-1(vt21)* and *dat-1(vt22)*, we assayed the response of *vt25* and *vt29* to increasing doses of exogenous DA. Relative to N2, all *dat-1* mutants, as well as *vt29*, exhibited a statistically significant, twofold increase in sensitivity to exogenous DA on solid substrate. In contrast, *vt25* demonstrated a more N2-like response to DA, our first indication that these genes support DA signaling via separate pathways. The molecular mechanisms supporting a hypersensitivity to exogenous DA are yet to be determined, though in the case of the *dat-1* mutants, a plausible basis is a reduced requirement for exogenous DA to achieve DOP-3 activation in animals that cannot capitalize on the DA clearance activity provided by DAT-1. This mechanism has some support from the demonstration that DAT-1 can accumulate other exogenous substrates, such as the dopaminergic toxin 6-OHDA (Nass *et al.* 2005). A second possibility is that, in DAT-1−deficient animals, chronically elevated levels of extrasynaptic DA may lead to a desensitization of a DA autoreceptor that may normally act to suppress DA release via a negative-feedback mechanism. In mammals, the D2 receptor serves both to suppress DA neuron firing and to reduce DA release (Usiello *et al.* 2000; Schmitz *et al.* 2002; Beckstead *et al.* 2004; Bello *et al.* 2011). Although such a mechanism has not been defined in nematodes, a D2-like DA receptor DOP-2 is also expressed by *C. elegans* DA neurons (Suo *et al.* 2003). By inference, desensitization of presynaptic DOP-2 produced by constitutively elevated extracellular DA could result in enhanced DA release that synergizes with exogenous DA, thereby reducing the amount of DA needed on plates needed to produce immobility. Studies are needed in which investigators use lines with DA neuron-specific elimination of DOP-2 to test this possibility. Such animals would also be useful in testing whether mutants recovered in our screen produce their hyperdopaminergic phenotype by suppressing DOP-2 mediated, inhibitory control of DA neuron excitability and/or DA release.

As our studies progressed, we recognized that our automated movement analysis software (Tracker) could be augmented to provide a much more detailed analysis of activity patterns than is reported in standard thrashing assays. To accomplish this goal, we developed a new suite of analytical tools that would allow for an unbiased assessment of the behavior of animals, both as individuals and across a population. Although the essential patterns among the strains we assayed were consistent, finer aspects of their swimming behavior were now observable. One such novel observation concerns the finding that a significant fraction of *dat-1* animals spontaneously recovered from paralysis for short intervals (termed here “reversals”). Because we observe these reversals in the context of a hyperdopaminergic phenotype, it is possible that excess postsynaptic DA signaling produces reversals in the course of moving between two distinct behavioral states. In this regard, Vidal-Gadea *et al.* 2011 demonstrated that DA is necessary and sufficient for *C. elegans* transit from swimming to crawling.

Whereas the Swip of *dat-1* animals is dependent solely on DOP-3, the swim-crawl transition requires DOP-1 and DOP-4 (Vidal-Gadea *et al.* 2011). Alternatively, reversals may derive from recruitment of other circuits that stimulate movement and that become evident when DOP-3 receptors are desensitized. Regardless, the relative presence of Swip revertants among our mutant strains suggests that these reversions can be triggered or unmasked by specific genetic perturbations in DA signaling. Specifically, we found that *vt29* exhibits a significantly reduced reversion incidence from *dat-1* and *vt25*. Multiple automated tracking systems for *C. elegans* swimming have been developed (Buckingham and Sattelle 2008; Ghosh and Emmons 2008; Pierce-Shimomura *et al.* 2008; Fang-Yen *et al.* 2010; Ghosh and Emmons 2010). Our system and its associated analytical tools (J. A. Hardaway and J. Wing, unpublished data) has been designed to detect multiple parameters of swimming behavior and subtle fluctuations between mobile and immobile states.

Our automated thrashing analysis of *vt25* and *vt29* revealed striking differences between them and with *dat-1*. Using SwimR, we observed that fewer *vt25* animals fail to paralyze than *dat-1* and that *vt29* has a shorter latency to paralyze and a lower incidence of Swip reversion from the *dat-1* strain. Reversion analysis also revealed that incidence of reversion is more sensitive to genotype than reversion frequency (no. reversals/animal), time to first reversion, or average reversion event length. These findings reveal that *vt29* differs from *dat-1* and *vt25* in its threshold for reversion and that behavior during reversion, as well as transition back to a paralytic state, is determined by independent factors. Based on additional functional data regarding the sensitivity of *vt29* to osmolarity, we propose that the biological mechanism impacted by *vt29* may determine overall sensitivity to the physical environment encountered in the Swip assay, whereas *vt25*, like *dat-1*, impacts mechanisms more directly supporting DA inactivation.

The kinetic differences in Swip behavior observed for *vt25* and *vt29* raised the question as to whether the strength of Swip in these lines is maintained under conditions in which Swip is suppressed in the *dat-1* strain. Previous study of the environmental dependence of the *dat-1* Swip phenotype revealed that an increase in the osmolarity of the swimming media significantly suppresses the Swip phenotype (J. A. Hardaway, personal communication to Worm Breeder's Gazette). In this study, we were able to replicate that finding by using both manual assays and automated thrashing analysis. Remarkably, *vt25* maintained its Swip behavior across all osmolarities, whereas *vt29* swimming behavior, like *dat-1*, was recovered as the osmolarity of the medium approached that of M9. We also assayed the behavior of the *dat-1*; *vt29* double mutant across these osmolarities and observed an increase in Swip penetrance at intermediate osmolarities.

These findings suggest that *dat-1* and *vt29* act in parallel, independent pathways that can sum to produce an additive paralytic response at intermediate osmolarities. Although both the *dat-1* and *vt29* strain demonstrated recovery of swimming in the isotonic buffer M9, our automated analysis demonstrated that, nonetheless, *dat-1* behavior could be differentiated from *vt29* in that the latter line maintains a stronger Swip phenotype even in media of high osmolarity. It is commonplace to use salt-buffered solutions in the study of neurotransmitter regulation of swimming, despite the fact that the organism normally resides in an environment with variable ionic content. Together, these studies demonstrate that the attribution of genes to DA signaling pathways, as well as their epistasis, will benefit from the use of osmotic profiling.

To investigate the potential pathways in which *vt25* and *vt29* reside, we assayed their Swip phenotypes in the context of DAT-1 transgenic overexpression, This transgene fully rescues the *dat-1* mutant (McDonald *et al.* 2007) and almost completely restored the swimming behavior of *vt25*, although short transient bouts were still evident. In contrast, *vt29* swimming behavior was only partially restored by DAT-1 overexpression. Because the *vt25* strain displays a weaker phenotype, the relative magnitude of suppression between *vt25* and *vt29* is roughly equivalent and suggests that overexpression of DAT can partially restore disruptions in multiple pathways regulating DA homeostasis. Manipulation of multiple DAT-interacting proteins have been implicated in the folding, trafficking, stability, and activity of the transporter, including PICK1 (Torres *et al.* 2001), Hic-5 (Carneiro *et al.* 2002), syntaxin1A (Lee *et al.* 2004), RACK1 (Lee *et al.* 2004), α-synuclein (Lee *et al.* 2001), synaptogyrin-3 (Egaña *et al.* 2009), and the D2 DA receptor (Lee *et al.* 2007), although the significance of these interactions *in vivo* has not been studied. Mutation of the *C. elegans* orthologs (where they exist) of these genes is unlikely to contribute to the phenotypes of *vt25* and *vt29* based on their absence from mapped loci. In addition, because we observed no difference in the somatic export of GFP:DAT-1, we doubt they produce Swip through alterations in transporter trafficking to the synapse. This is particularly evident with *vt29* animals that retained the full 6-OHDA sensitivity exhibited by N2. However, *vt25* displayed a more moderate 6-OHDA sensitivity and thus part of its actions may involve local, synaptic DAT-1 trafficking to the plasma membrane or control of transporter activity.

Finally, as *vt25* or *vt29* display a hyperdopaminergic signaling phenotype, release of enhanced, synthesized, or stored DA could explain their phenotypes. However, when we measured the DA content of these lines by HPLC, we found that, as with *dat-1*, both exhibited a significant *decrease* in DA levels, although not as reduced as the *cat-2* mutant that actually fails to Swip. Measurement of synaptic DA *in vivo* in *C. elegans* is not currently feasible. Interestingly, however, DAT KO mice demonstrate tonically elevated synaptic DA, accompanied by a reduction in tissue DA (Giros *et al.* 1996), which these investigators ascribed to a failure to replenish resting vesicular stores via recapture of synaptic DA. Although, as noted previously, a contribution of *vt25* to DAT trafficking or activity could result in reduced DA levels, multiple lines of evidence do not support such a role for *vt29*. Rather we hypothesize that the gene mutated in *vt29* causes excess DA release that cannot be restored through the normal pathways of DA synthesis and recapture. A reasonable hypothesis for the phenotype of the *vt29* mutant (and to a lesser degree *vt25*), and one that now can be targeted in future studies, is that the *vt29* gene normally exerts suppression of DA neuron excitability and/or DA vesicular release (Figure 6G). Efforts are underway to identify the sites of the molecular lesions borne by *vt25* and *vt29* animals. The conservation of components of DA signaling throughout phylogeny suggests that our efforts in this regard may identify molecules whose intensive study can provide insights into brain disorders involving compromised DA signaling, as well as the development of novel therapeutics.

## Supplementary Material

Supporting Information
