# Supplementary material for: Forward Genetic Analysis to Identify Determinants of Dopamine Signaling in Caenorhabditis elegans Using Swimming-Induced Paralysis
Source: G3 (Bethesda). 2012 Aug 1;2(8):961–75. doi: 10.1534/g3.112.003533 (PMC3411251; doi:10.1534/g3.112.003533)
Supplement: Supporting Information [file supp_2_8_961__index.html]

Supporting Information 

# Forward Genetic Analysis to Identify Determinants of Dopamine Signaling in *Caenorhabditis elegans* Using Swimming-Induced Paralysis

## Supporting Information for Hardaway *et al.*, 2012

**Files in this Data Supplement:**

- Supporting Information - Figures S1-S7 (PDF, 4.8 MB)
- Figure S1 - *Swip* mutants possess normal DA neuron morphology (PDF, 890 KB)
- Figure S2 - Complementation assays of *swip* strains to N2 and *dat-1* (PDF, 195 KB)
- Figure S3 - Modeling and Functional Analyses of *dat-1* mutations (PDF, 1.6 MB)
- Figure S4 - *vt25* and *vt29* map to LGIII and LGX respectively (PDF, 376 KB)
- Figure S5 - Behavioral Analyses of the *dat-1(vt21)* and *dat-1(vt22)* strains (PDF, 1.3 MB)
- Figure S6 - Histogram of automated thrashing behavior in N2, *dat-1(ok157)* and *swip* lines generated by SwimR software. (PDF, 350 KB)
- Figure S7 - *vt25* and *vt29* do not alter GFP-DAT somatic export (PDF, 454 KB)
