# Supplementary material for: Forward Genetic Analysis to Identify Determinants of Dopamine Signaling in Caenorhabditis elegans Using Swimming-Induced Paralysis
Source: G3 (Bethesda). 2012 Aug 1;2(8):961–75. doi: 10.1534/g3.112.003533 (PMC3411251; doi:10.1534/g3.112.003533)
Supplement: Supporting Information [file supp_2.8.961_003533SI.pdf]

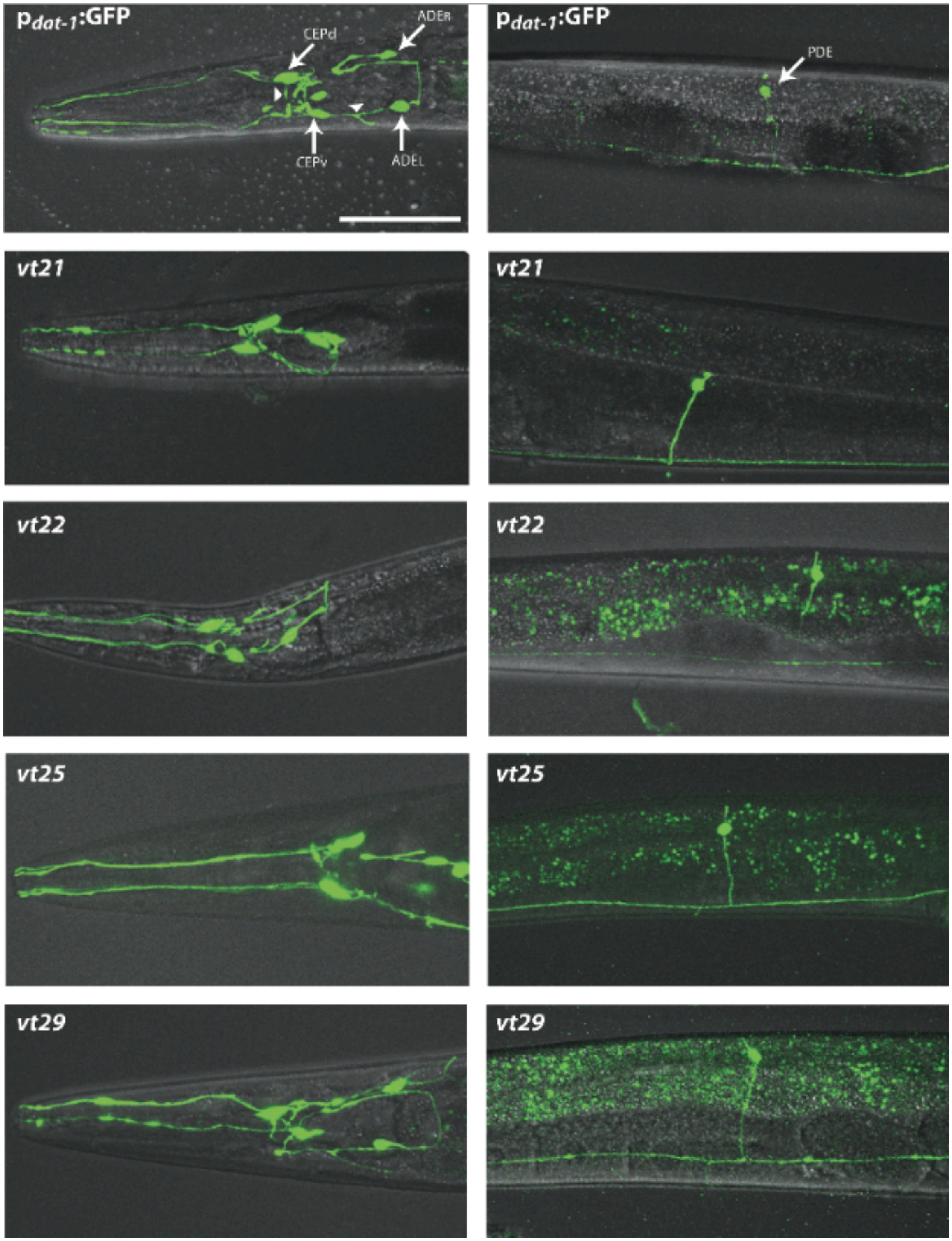

Figure S1 *Swip* mutants possess normal DA neuron morphology. The left panels show that CEP and ADE neurons and processes are intact in all strains, possessing visibly normal dendrites(arrows) and terminals(arrowheads). On the right, PDE neurons and projections are shown for each strain, where a normal morphology is also evident. For all strains used, fully outcrossed *vt21, vt22,* vt25 *and vt29* were crossed onto a strain bearing an integrated p$_{dat-1}$:GFP transgene(BY250, *vtIs7*). *Swip* mutant genotypes were confirmed by Swip behavioral tests as the *vtIs7* line shows no paralysis on its own. Anterior is left in all images shown. Scale bar is equal to 50 μM.

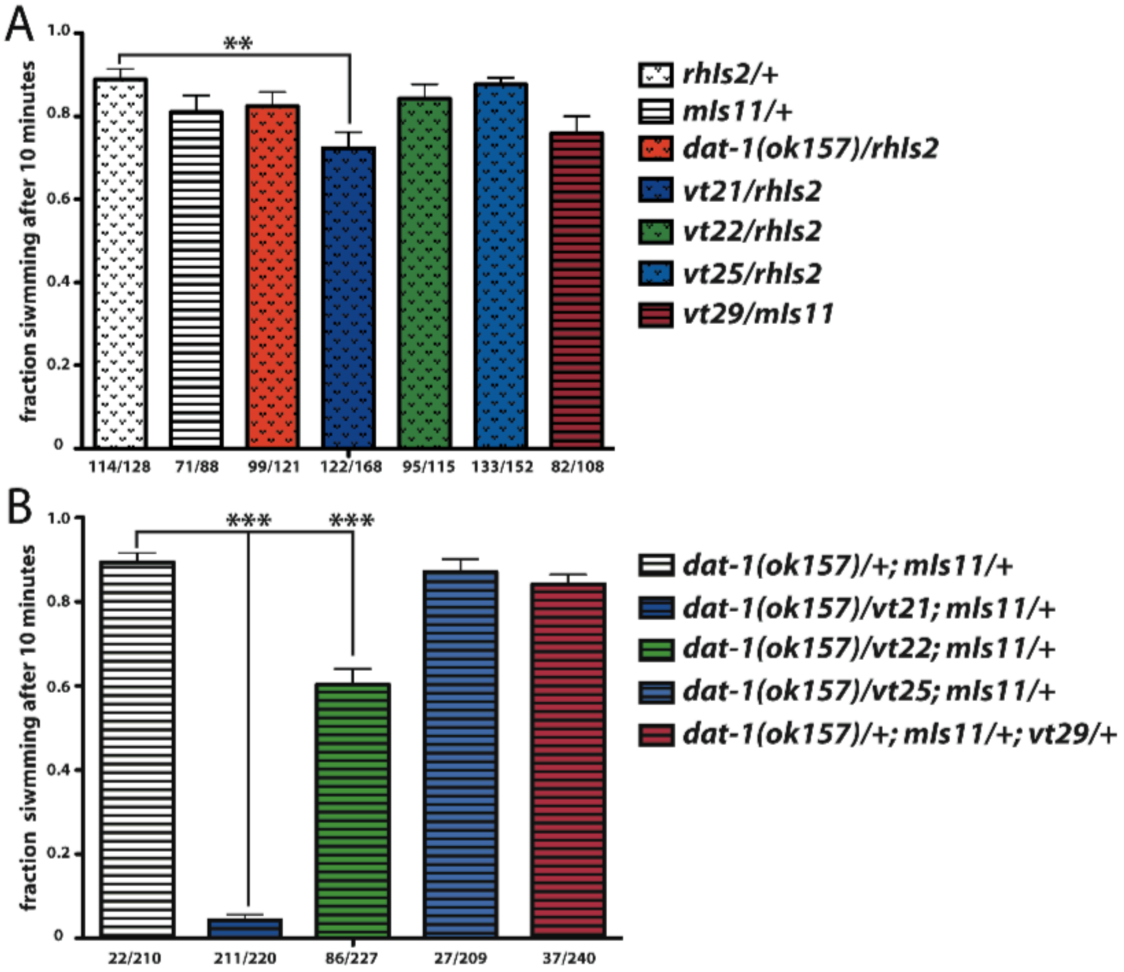

Figure S2 Complementation assays of *swip* strains to N2 and *dat-1.* **A,** *swip* mutant heterozygotes are fully rescued in their Swip behavior. *vt21/rhIs2* does show a significant reduction from control levels, but Swip is greatly suppressed from that seen in homozygotes (see Fig. 2). Lines containing integrated fluorescent transgenes were crossed to fully outcrossed *swip* hermaphrodites and fluorescent cross progeny were tested for Swip. These balancer lines display normal swimming behavior on their own. Data were analyzed using one-way ANOVA with selected Bonferroni post-tests comparing to controls bearing a single copy fluorescent transgene. Fractions below the bars represent # paralyzed/# assayed. **B**, *vt21* and *vt22* fail to complement *dat-1*, whereas *vt25* and *vt29* suppress Swip to control levels. *Dat-1(ok157)* males bearing a fluorescent transgene *in trans* were crossed to fully outcrossed *swip* mutants and fluorescent cross progeny were tested for Swip. Data were analyzed as in **A**.

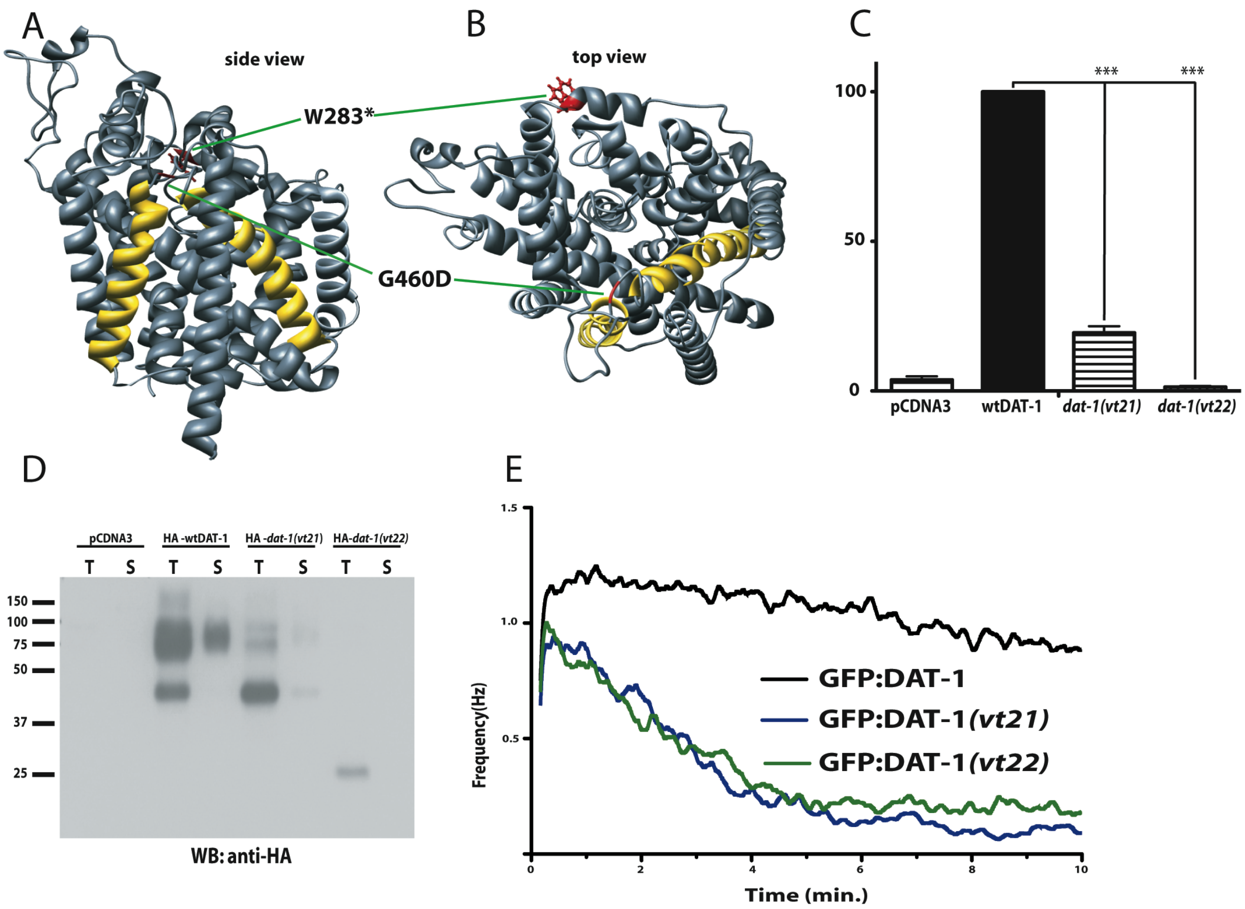

Figure S3 Modeling and Functional Analyses of *dat-1* mutations. **A** and **B.** Positions of *vt21* and *vt22* mutations on DAT-1 protein, as predicted from the solved crystal structure of the bacterial leucine transporter ($LeuT_{Aa}$). Mutant residues are shown in red and TMs 9 and 10 adjoining the *vt22* mutation are colored in yellow. **A** – side view, **B** – extracellular view. **C**. *Vt21* and *vt22* (*dat-1(vt21)* and *dat-1(vt22*), respectively) exhibit reduced DA transport activity *in vitro*. COS-7 cells were transiently transfected with either empty vector (pcDNA3), or constructs expressing DAT-1(pRB606), DAT-1*(vt21)*(pRB1026) or DAT-1*(vt22)*(pRB1027) proteins and assayed for DA transport activity as described in Methods. Both of the mutant DAT-1 proteins yielded significantly reduced transport activity as compared to WT DAT-1. Values represent the mean % DA uptake of WT +/- SEM of six independent experiments and were compared using one-way ANOVA with Bonferroni post tests to WT. ****P*<0.001. **D.** Mutant DAT-1 proteins display altered levels and trafficking of transporter protein. Total and surface protein expression of HA-tagged DAT-1(pRB491), DAT-1*(vt21)*(pRB1028) and DAT-1*(vt22)*(pRB1029) were determined by western blot analysis, as described in Methods. Wildtype DAT-1 expression is evident as an immature species of ~45 kDa and a mature, glycosylated band at ~ 80 kDa, with the 80 kDa species detected in surface fractions. DAT-1*(vt21)* expression is detected as both an immature and a full length species, with a higher relative abundance of the immature species. Little to no expression of these species is detected in surface fractions. No full length product is evident in the total or surface lysates from DAT-1*(vt22)* transfected cells with only a short ~25 kDa fragment evident, consistent with the site of the nonsense mutation. Image presented is representative of 4 independent experiments with equivalent results. **E.** *vt21* and *vt22* mutations, engineered into GFP:DAT-1 expression constructs, fail to rescue Swip behavior of *dat-1(ok157);lin-15(n765ts)* animals. In contrast, expression of GFP:DAT-1 fully rescues Swip. Behavior plotted of animals expressing GFP:DAT-1(wt) is the average of four independent transgenic lines. Data from *vt21* and *vt22* mutant GFP:DAT-1 lines derives from at least 20 animals, with three lines scored for each test. Traces were compared using two-way ANOVA and multiple Bonferroni posttests where swimming behavior of GFP:DAT-1 fusions bearing *vt21* and *vt22* mutations was significantly reduced from WT (p<0.001 for all values along the running average after the first minute of swimming). The behavior of the two mutants was not significantly different from each other.

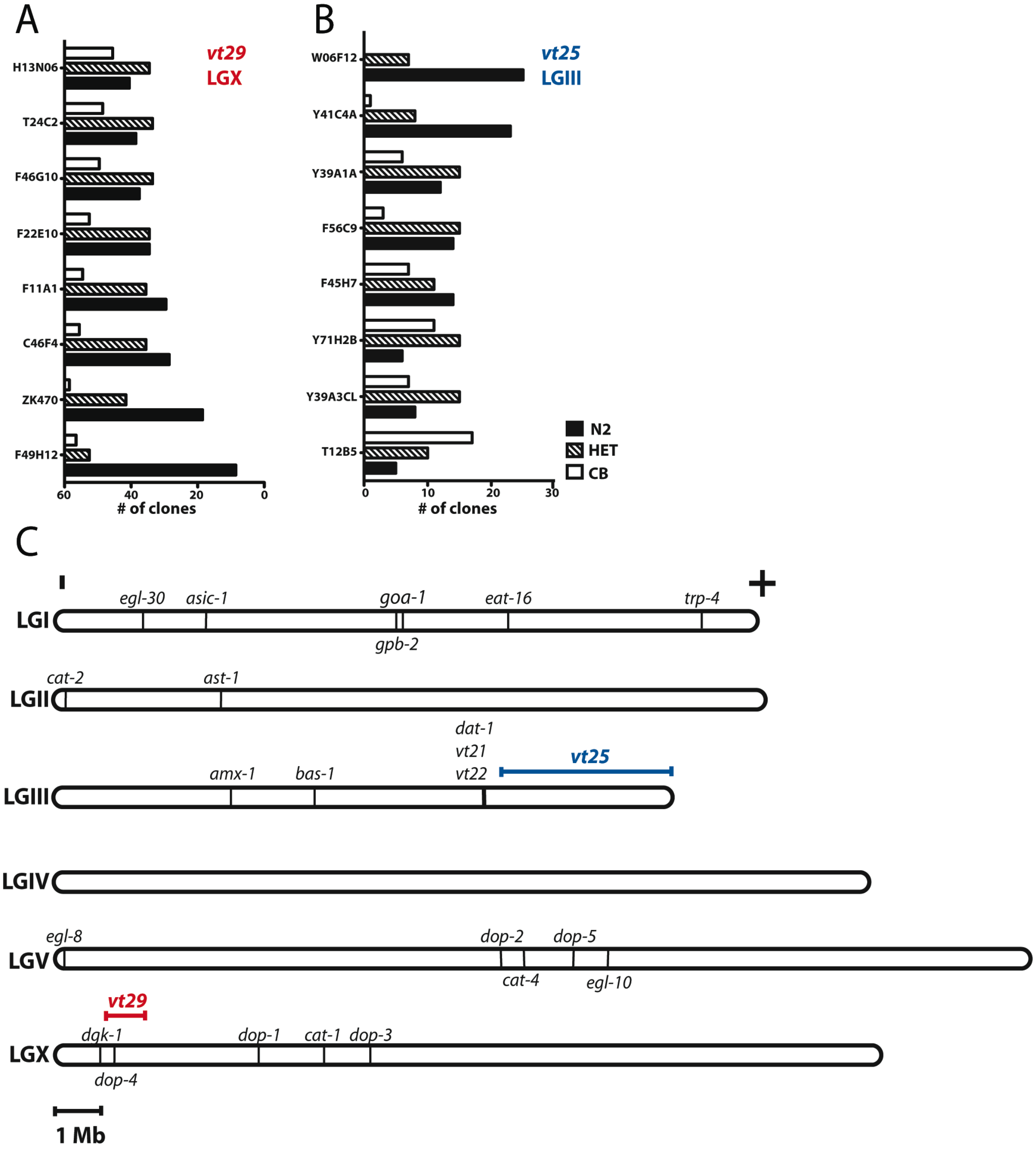

Figure S4 *vt25* and *vt29* map to LGIII and LGX respectively. **A + B.** For *vt25*, a Bristol Island was found on LGIII closest to the W06F12 SNP, and on LGX closest to the ZK470 SNP. Linkage was first demonstrated with bulk segregant mapping to LGIII and LGX, after which we used fine mapping to generate the plots above. A more detailed protocol is described in the Methods section. **C.** Genomic map of known genes regulating DA signaling in *C. elegans*. After a literature search, positions of known genes were used to build a map of genomic loci that act in dopaminergic pathways. Chromosomes, gene positions and mapping locations are drawn to scale. *vt25* maps to a region with no other known loci, while *vt29* maps to a region proximal to *dgk-1* and containing *dop-4*. *vt29* does not contain mutations in these two genes.

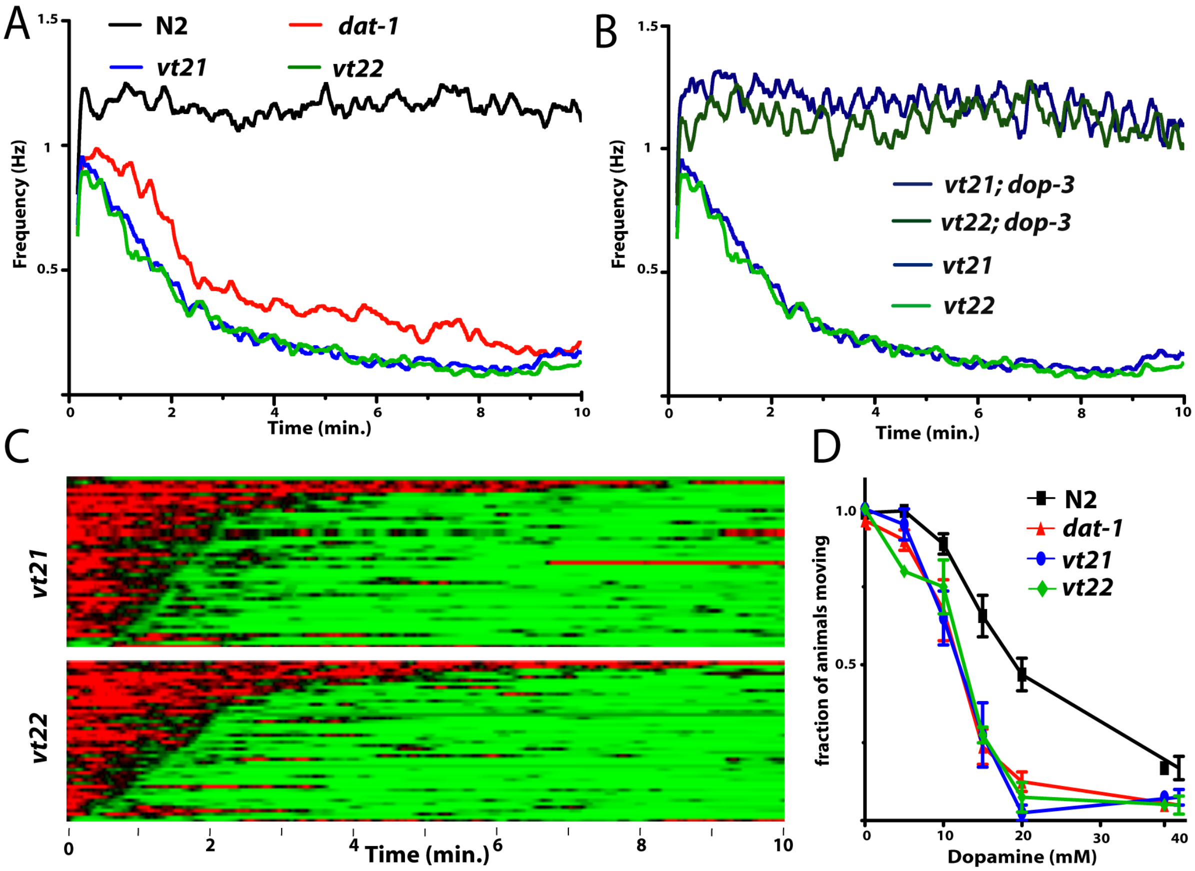

Figure S5 Behavioral Analyses of the *dat-1(vt21)* and *dat-1(vt22)* strains. **A.** *Vt21* and *vt22* mimic the *dat-1* Swip phenotype as measured by automated thrashing analysis. Individual animals were recorded using a video capture system and then analyzed with customed-designed Thrasher software that assigns multiple linear elements projecting from the worm centroid. The position of these linear elements are tracked and converted off-line to movement frequency as a function of time. Batch conversions are generated, providing mean values and SEM along moving averages. Error bars are not shown in these plots for simplicity. *Dat-1(ok157)*, *vt21* and *vt22* were found to be significantly different from N2 using two-way ANOVA with Bonferroni posttests of mutants to N2, with each mutant possessing a $P<0.001$ after the one minute mark. **B.** Mutation of the postsynaptic receptor DOP-3 fully rescues the paralysis phenotype of *vt21* and *vt22*. Analyses were performed as described in **B**, where *vt21* and *vt22* were both found to be significantly different from the double mutants *vt21;dop-3(vs106)* and *vt22;dop-3(vs106)* with $P< 0.001$ after 1 minute. **C.** Heat map representations of *dat-1(vt21)* and *dat-1(vt22)* swimming traces. Analyses were performed as described in Figure 4. **D.** *vt21* and *vt22* display enhanced sensitivity to exogenous DA when tested on solid medium, as compared to N2, but are indistinguishable from *dat-1*. For these assays, 10 L4 stage worms were placed on plates containing increasing concentrations of exogenous DA, incubated for 20 min and then scored for 10 sec as paralyzed or moving. Dose-response curves were compared using two-way ANOVA with Bonferroni posttests comparing mutants to N2, in which *dat-1*, *vt21* + *vt22* were all found to be significantly different from N2, with a $P<0.001$ at 15 and 20 mM DA. Data derive from at least 4 tests per strain per DA concentration. Error bars represent SEM. Exogenous DA dose response profiles and data analysis were performed as described in the Methods and in Figure 3.

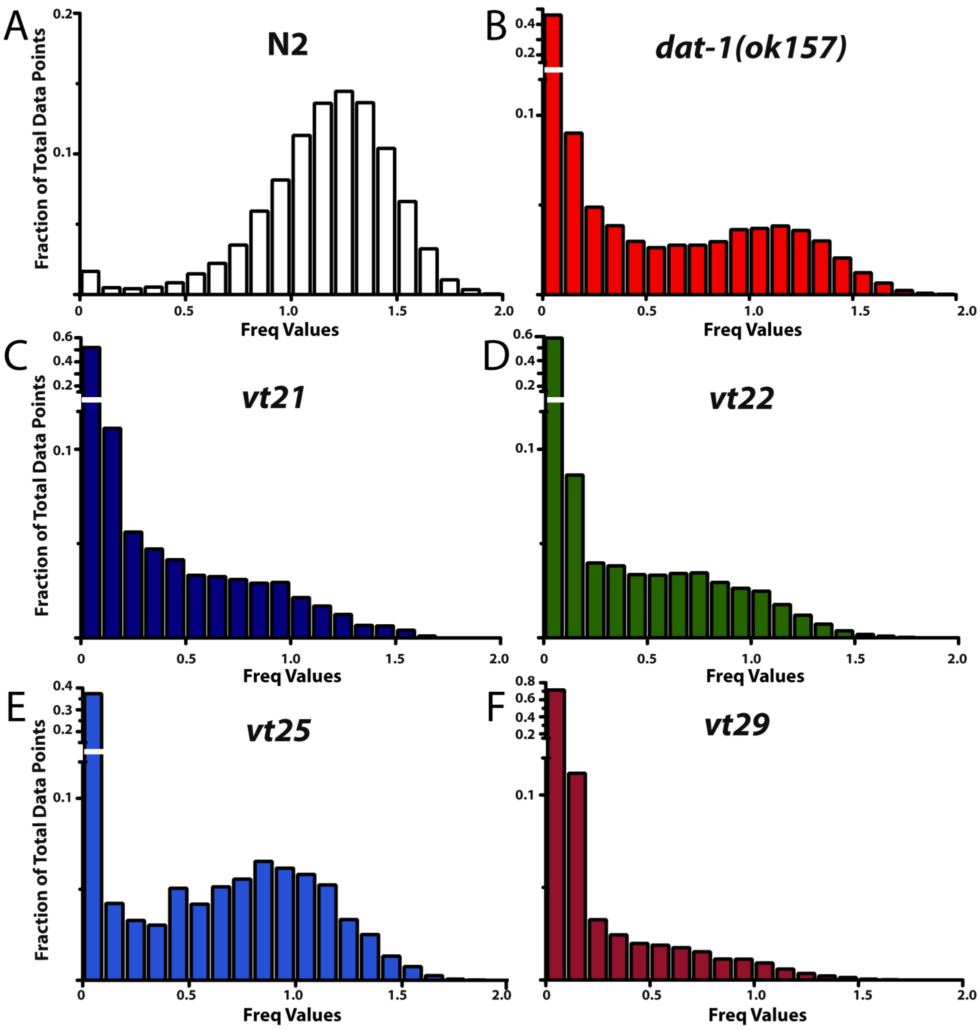

Figure S6 Histogram of automated thrashing behavior in N2, *dat-1(ok157)* and *swip* lines generated by SwimR software. For all plots (**A-F**), the total # of data points for all animals within a genotype were grouped into successive 0.1 Hz bins and plotted as the fraction of the total # of data points. In the *swip* mutants (**C-F**), the y-axis and first column are broken in order to visualize the patterns of lower frequency bins.

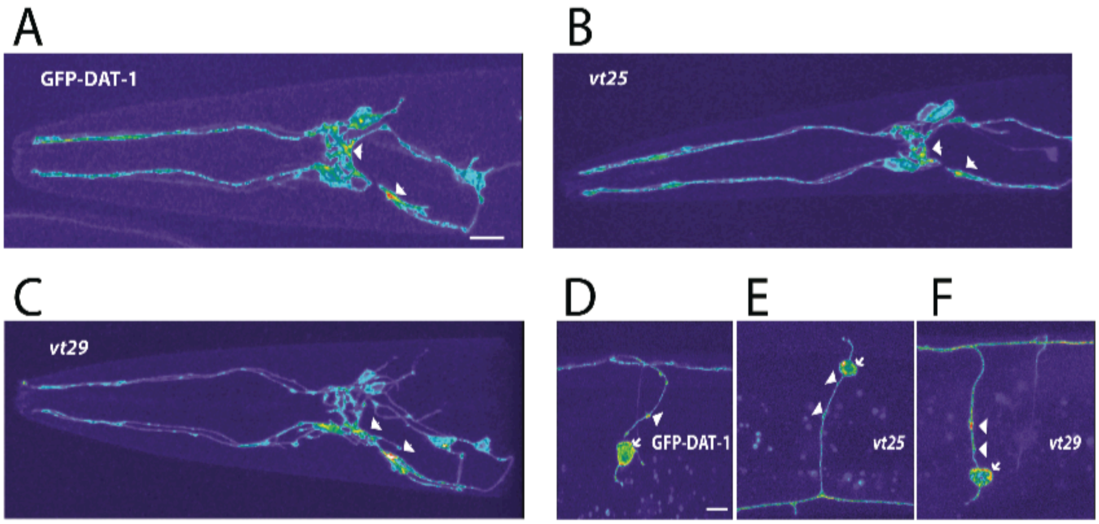

Figure S7 *vt25* and *vt29* do not alter GFP-DAT somatic export: Expression pattern of p_{dat-1}::GFP:DAT-1(*vtIs18)* in the head(CEP and ADE neurons, **A,B + C**) and posterior(PDE, **D,E + F**) in WT, *vt25* and *vt29* were analyzed by confocal microscopy. The expression pattern of GFP:DAT-1 is not altered on the *vt25* or *vt29* backgrounds, with diffuse expression in DA neuron cell bodies(arrows) and punctate expression visible at terminal regions (arrowheads). Scale bar equal 10 μM.
