# Supplementary material for: Forward Genetic Analysis to Identify Determinants of Dopamine Signaling in Caenorhabditis elegans Using Swimming-Induced Paralysis
Source: G3 (Bethesda). 2012 Aug 1;2(8):961–75. doi: 10.1534/g3.112.003533 (PMC3411251; doi:10.1534/g3.112.003533)
Supplement: Supporting Information [file supp_2.8.961_FigureS1.pdf]

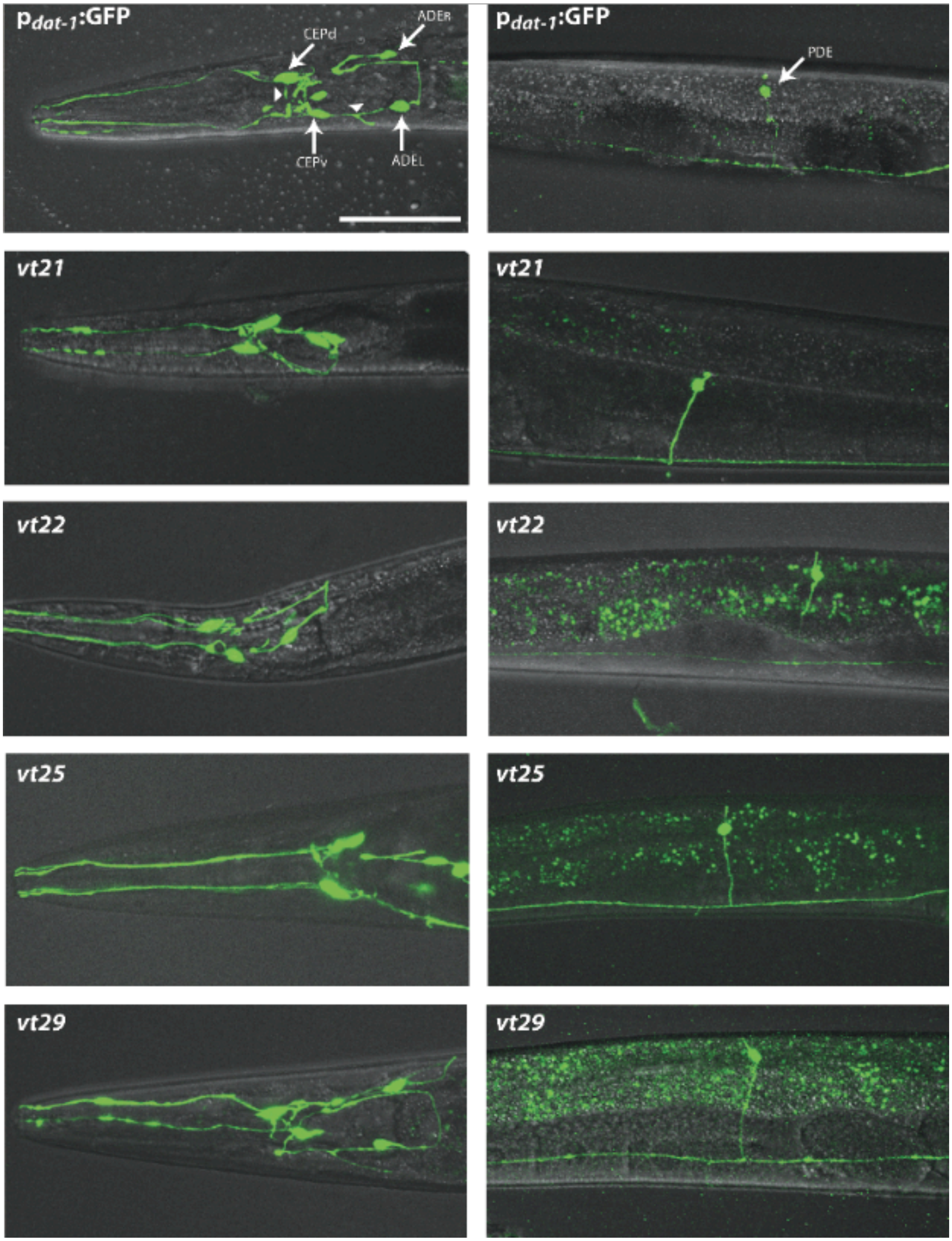

Figure S1 *Swip* mutants possess normal DA neuron morphology. The left panels show that CEP and ADE neurons and processes are intact in all strains, possessing visibly normal dendrites(arrows) and terminals(arrowheads). On the right, PDE neurons and projections are shown for each strain, where a normal morphology is also evident. For all strains used, fully outcrossed *vt21, vt22,* vt25 *and vt29* were crossed onto a strain bearing an integrated p_{dat-1}:GFP transgene(BY250, *vtIs7*). *Swip* mutant genotypes were confirmed by Swip behavioral tests as the *vtIs7* line shows no paralysis on its own. Anterior is left in all images shown. Scale bar is equal to 50 μM.
