# Supplementary material for: Forward Genetic Analysis to Identify Determinants of Dopamine Signaling in Caenorhabditis elegans Using Swimming-Induced Paralysis
Source: G3 (Bethesda). 2012 Aug 1;2(8):961–75. doi: 10.1534/g3.112.003533 (PMC3411251; doi:10.1534/g3.112.003533)
Supplement: Supporting Information [file supp_2.8.961_FigureS2.pdf]

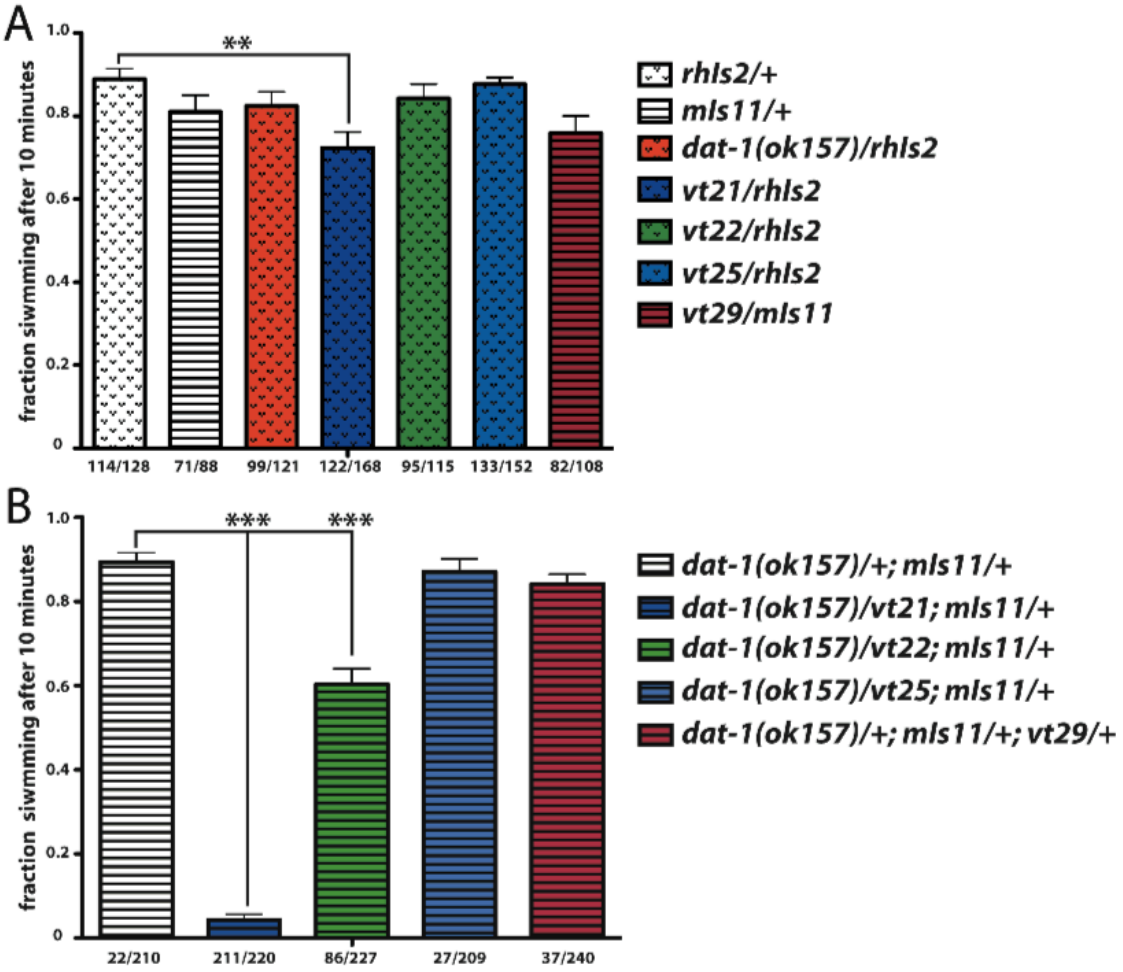

Figure S2 Complementation assays of *swip* strains to N2 and *dat-1.* **A,** *swip* mutant heterozygotes are fully rescued in their Swip behavior. *vt21/rhIs2* does show a significant reduction from control levels, but Swip is greatly suppressed from that seen in homozygotes (see Fig. 2). Lines containing integrated fluorescent transgenes were crossed to fully outcrossed *swip* hermaphrodites and fluorescent cross progeny were tested for Swip. These balancer lines display normal swimming behavior on their own. Data were analyzed using one-way ANOVA with selected Bonferroni post-tests comparing to controls bearing a single copy fluorescent transgene. Fractions below the bars represent # paralyzed/# assayed. **B**, *vt21* and *vt22* fail to complement *dat-1*, whereas *vt25* and *vt29* suppress Swip to control levels. *Dat-1(ok157)* males bearing a fluorescent transgene *in trans* were crossed to fully outcrossed *swip* mutants and fluorescent cross progeny were tested for Swip. Data were analyzed as in **A**.
