# Supplementary material for: Forward Genetic Analysis to Identify Determinants of Dopamine Signaling in Caenorhabditis elegans Using Swimming-Induced Paralysis
Source: G3 (Bethesda). 2012 Aug 1;2(8):961–75. doi: 10.1534/g3.112.003533 (PMC3411251; doi:10.1534/g3.112.003533)
Supplement: Supporting Information [file supp_2.8.961_FigureS3.pdf]

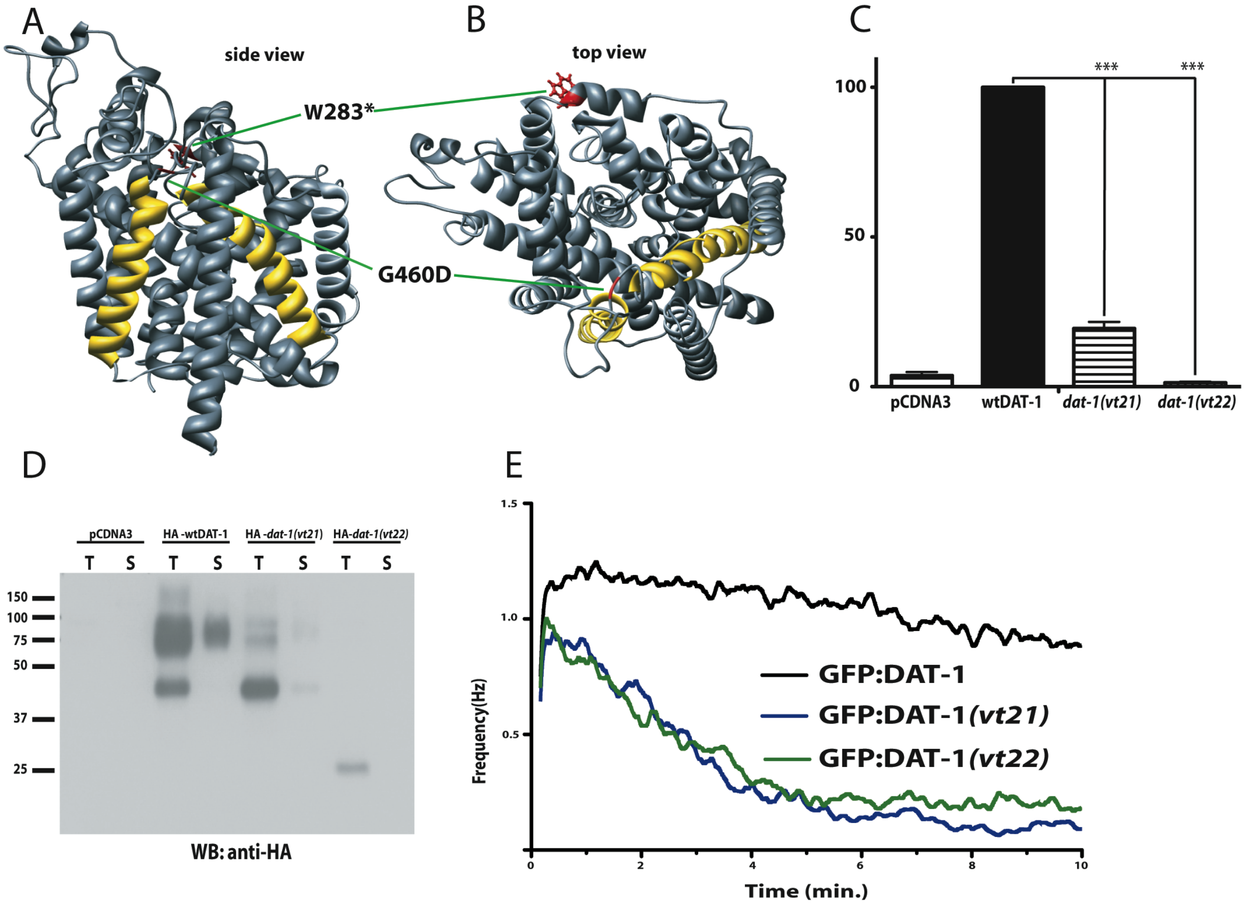

Figure S3 Modeling and Functional Analyses of *dat-1* mutations. **A** and **B.** Positions of *vt21* and *vt22* mutations on DAT-1 protein, as predicted from the solved crystal structure of the bacterial leucine transporter ($LeuT_{Aa}$). Mutant residues are shown in red and TMs 9 and 10 adjoining the *vt22* mutation are colored in yellow. **A** – side view, **B** – extracellular view. **C**. *Vt21* and *vt22* (*dat-1(vt21)* and *dat-1(vt22*), respectively) exhibit reduced DA transport activity *in vitro*. COS-7 cells were transiently transfected with either empty vector (pcDNA3), or constructs expressing DAT-1(pRB606), DAT-1*(vt21)*(pRB1026) or DAT-1*(vt22)*(pRB1027) proteins and assayed for DA transport activity as described in Methods. Both of the mutant DAT-1 proteins yielded significantly reduced transport activity as compared to WT DAT-1. Values represent the mean % DA uptake of WT +/- SEM of six independent experiments and were compared using one-way ANOVA with Bonferroni post tests to WT. ****P*<0.001. **D.** Mutant DAT-1 proteins display altered levels and trafficking of transporter protein. Total and surface protein expression of HA-tagged DAT-1(pRB491), DAT-1*(vt21)*(pRB1028) and DAT-1*(vt22)*(pRB1029) were determined by western blot analysis, as described in Methods. Wildtype DAT-1 expression is evident as an immature species of ~45 kDa and a mature, glycosylated band at ~ 80 kDa, with the 80 kDa species detected in surface fractions. DAT-1*(vt21)* expression is detected as both an immature and a full length species, with a higher relative abundance of the immature species. Little to no expression of these species is detected in surface fractions. No full length product is evident in the total or surface lysates from DAT-1*(vt22)* transfected cells with only a short ~25 kDa fragment evident, consistent with the site of the nonsense mutation. Image presented is representative of 4 independent experiments with equivalent results. **E.** *vt21* and *vt22* mutations, engineered into GFP:DAT-1 expression constructs, fail to rescue Swip behavior of *dat-1(ok157);lin-15(n765ts)* animals. In contrast, expression of GFP:DAT-1 fully rescues Swip. Behavior plotted of animals expressing GFP:DAT-1(wt) is the average of four independent transgenic lines. Data from *vt21* and *vt22* mutant GFP:DAT-1 lines derives from at least 20 animals, with three lines scored for each test. Traces were compared using two-way ANOVA and multiple Bonferroni posttests where swimming behavior of GFP:DAT-1 fusions bearing *vt21* and *vt22* mutations was significantly reduced from WT (p<0.001 for all values along the running average after the first minute of swimming). The behavior of the two mutants was not significantly different from each other.
