# Supplementary material for: Forward Genetic Analysis to Identify Determinants of Dopamine Signaling in Caenorhabditis elegans Using Swimming-Induced Paralysis
Source: G3 (Bethesda). 2012 Aug 1;2(8):961–75. doi: 10.1534/g3.112.003533 (PMC3411251; doi:10.1534/g3.112.003533)
Supplement: Supporting Information [file supp_2.8.961_FigureS4.pdf]

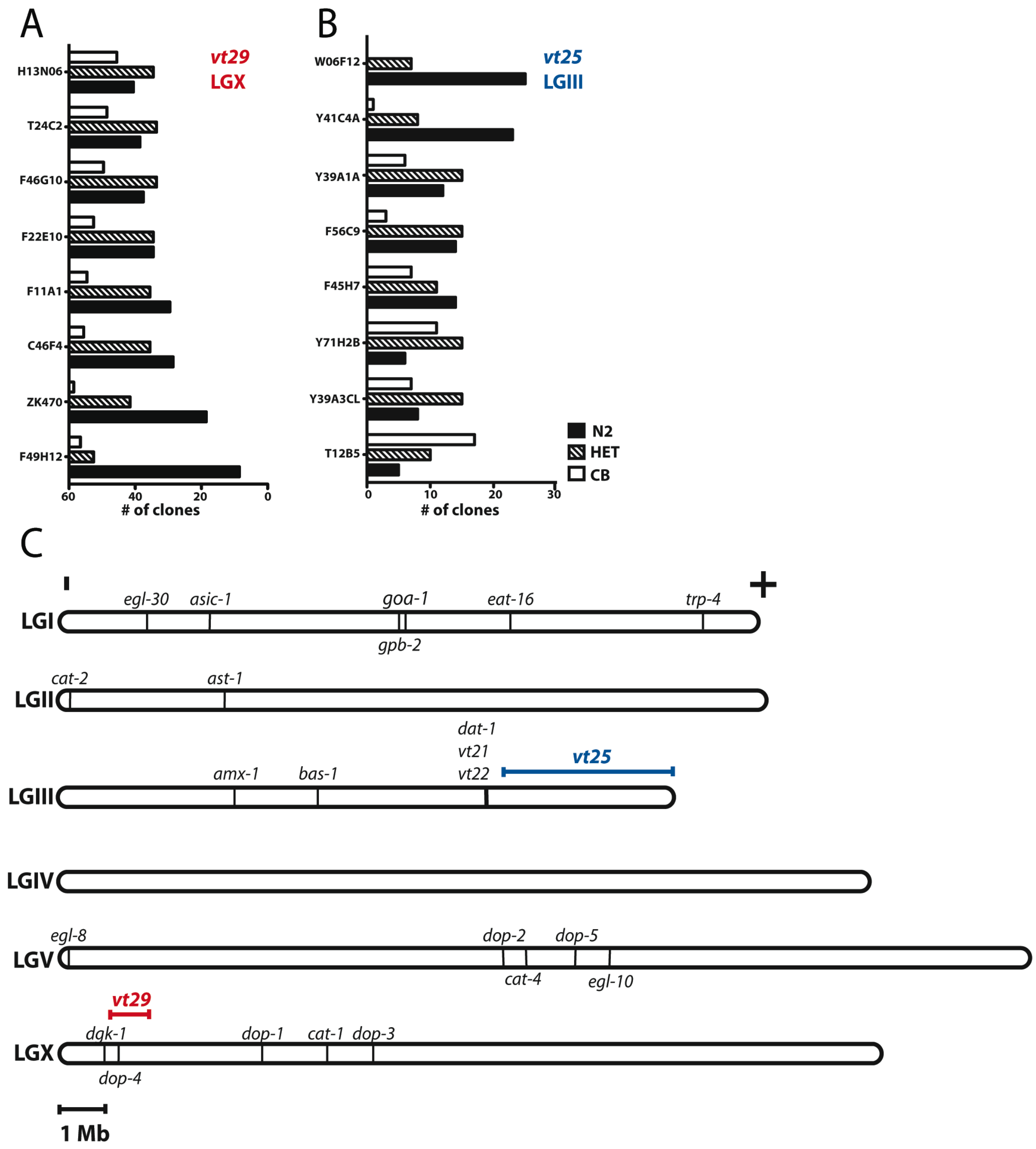

Figure S4 *vt25* and *vt29* map to LGIII and LGX respectively. **A + B.** For *vt25*, a Bristol Island was found on LGIII closest to the W06F12 SNP, and on LGX closest to the ZK470 SNP. Linkage was first demonstrated with bulk segregant mapping to LGIII and LGX, after which we used fine mapping to generate the plots above. A more detailed protocol is described in the Methods section. **C.** Genomic map of known genes regulating DA signaling in *C. elegans*. After a literature search, positions of known genes were used to build a map of genomic loci that act in dopaminergic pathways. Chromosomes, gene positions and mapping locations are drawn to scale. *vt25* maps to a region with no other known loci, while *vt29* maps to a region proximal to *dgk-1* and containing *dop-4*. *vt29* does not contain mutations in these two genes.
