# Supplementary material for: Forward Genetic Analysis to Identify Determinants of Dopamine Signaling in Caenorhabditis elegans Using Swimming-Induced Paralysis
Source: G3 (Bethesda). 2012 Aug 1;2(8):961–75. doi: 10.1534/g3.112.003533 (PMC3411251; doi:10.1534/g3.112.003533)
Supplement: Supporting Information [file supp_2.8.961_FigureS5.pdf]

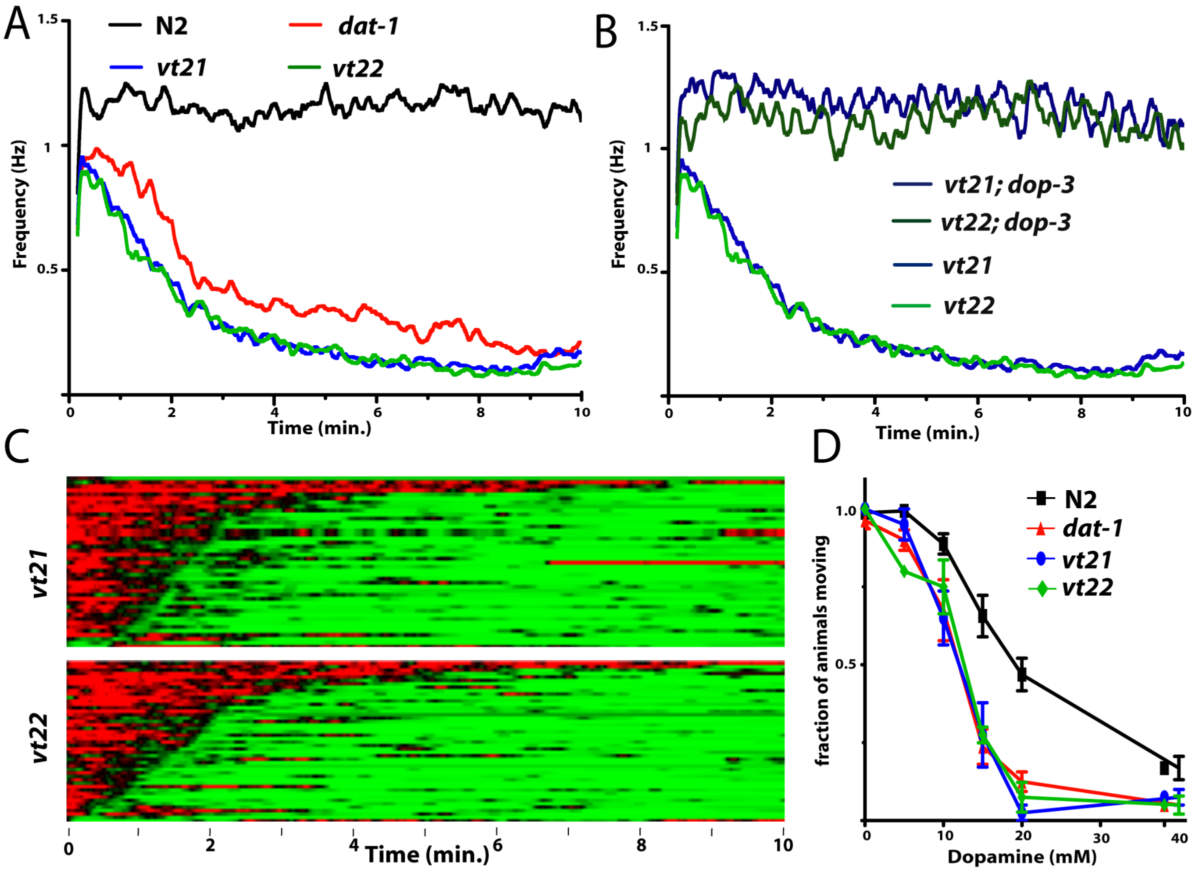

Figure S5 Behavioral Analyses of the *dat-1(vt21)* and *dat-1(vt22)* strains. **A.** *Vt21* and *vt22* mimic the *dat-1* Swip phenotype as measured by automated thrashing analysis. Individual animals were recorded using a video capture system and then analyzed with customed-designed Thrasher software that assigns multiple linear elements projecting from the worm centroid. The position of these linear elements are tracked and converted off-line to movement frequency as a function of time. Batch conversions are generated, providing mean values and SEM along moving averages. Error bars are not shown in these plots for simplicity. *Dat-1(ok157)*, *vt21* and *vt22* were found to be significantly different from N2 using two-way ANOVA with Bonferroni posttests of mutants to N2, with each mutant possessing a $P<0.001$ after the one minute mark. **B.** Mutation of the postsynaptic receptor DOP-3 fully rescues the paralysis phenotype of *vt21* and *vt22*. Analyses were performed as described in **B**, where *vt21* and *vt22* were both found to be significantly different from the double mutants *vt21;dop-3(vs106)* and *vt22;dop-3(vs106)* with $P< 0.001$ after 1 minute. **C.** Heat map representations of *dat-1(vt21)* and *dat-1(vt22)* swimming traces. Analyses were performed as described in Figure 4. **D.** *vt21* and *vt22* display enhanced sensitivity to exogenous DA when tested on solid medium, as compared to N2, but are indistinguishable from *dat-1*. For these assays, 10 L4 stage worms were placed on plates containing increasing concentrations of exogenous DA, incubated for 20 min and then scored for 10 sec as paralyzed or moving. Dose-response curves were compared using two-way ANOVA with Bonferroni posttests comparing mutants to N2, in which *dat-1*, *vt21* + *vt22* were all found to be significantly different from N2, with a $P<0.001$ at 15 and 20 mM DA. Data derive from at least 4 tests per strain per DA concentration. Error bars represent SEM. Exogenous DA dose response profiles and data analysis were performed as described in the Methods and in Figure 3.
