# Supplementary material for: Forward Genetic Analysis to Identify Determinants of Dopamine Signaling in Caenorhabditis elegans Using Swimming-Induced Paralysis
Source: G3 (Bethesda). 2012 Aug 1;2(8):961–75. doi: 10.1534/g3.112.003533 (PMC3411251; doi:10.1534/g3.112.003533)
Supplement: Supporting Information [file supp_2.8.961_FigureS6.pdf]

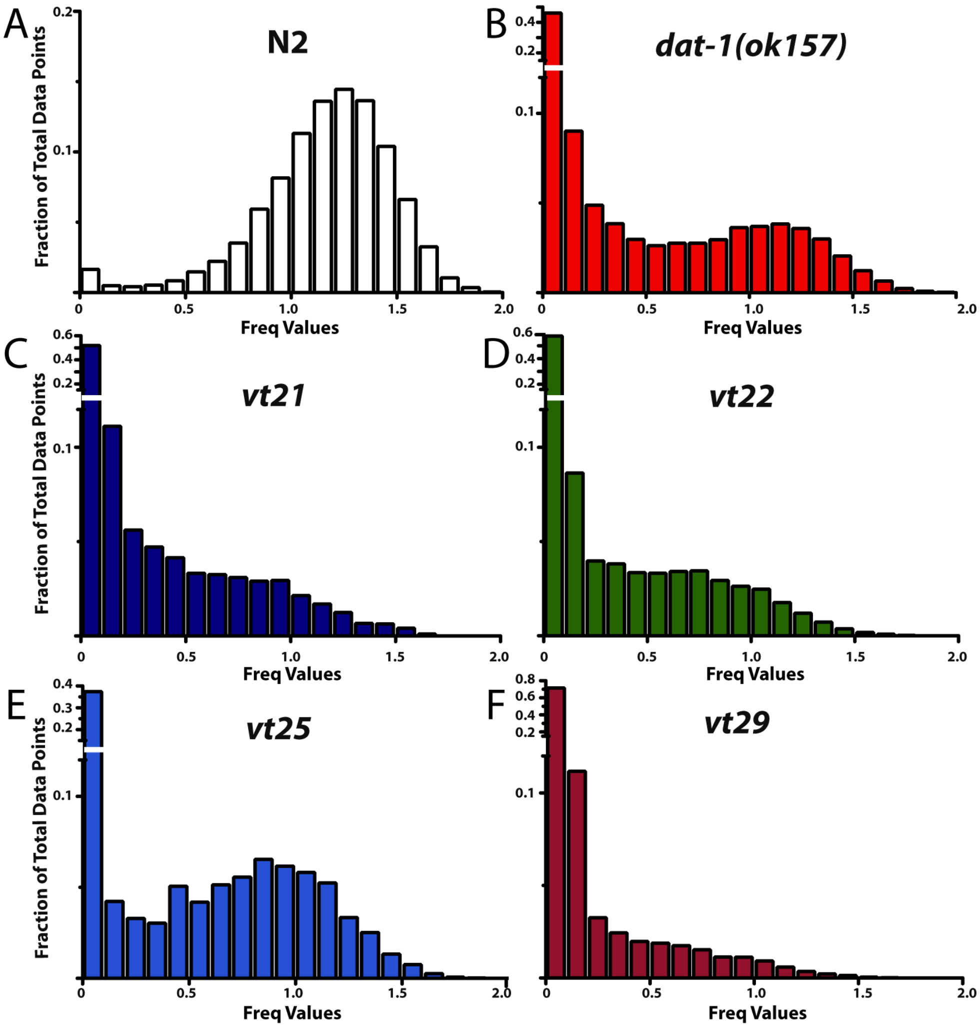

Figure S6 Histogram of automated thrashing behavior in N2, *dat-1(ok157)* and *swip* lines generated by SwimR software. For all plots (**A-F**), the total # of data points for all animals within a genotype were grouped into successive 0.1 Hz bins and plotted as the fraction of the total # of data points. In the *swip* mutants (**C-F**), the y-axis and first column are broken in order to visualize the patterns of lower frequency bins.
