# Supplementary material for: Forward Genetic Analysis to Identify Determinants of Dopamine Signaling in Caenorhabditis elegans Using Swimming-Induced Paralysis
Source: G3 (Bethesda). 2012 Aug 1;2(8):961–75. doi: 10.1534/g3.112.003533 (PMC3411251; doi:10.1534/g3.112.003533)
Supplement: Supporting Information [file supp_2.8.961_FigureS7.pdf]

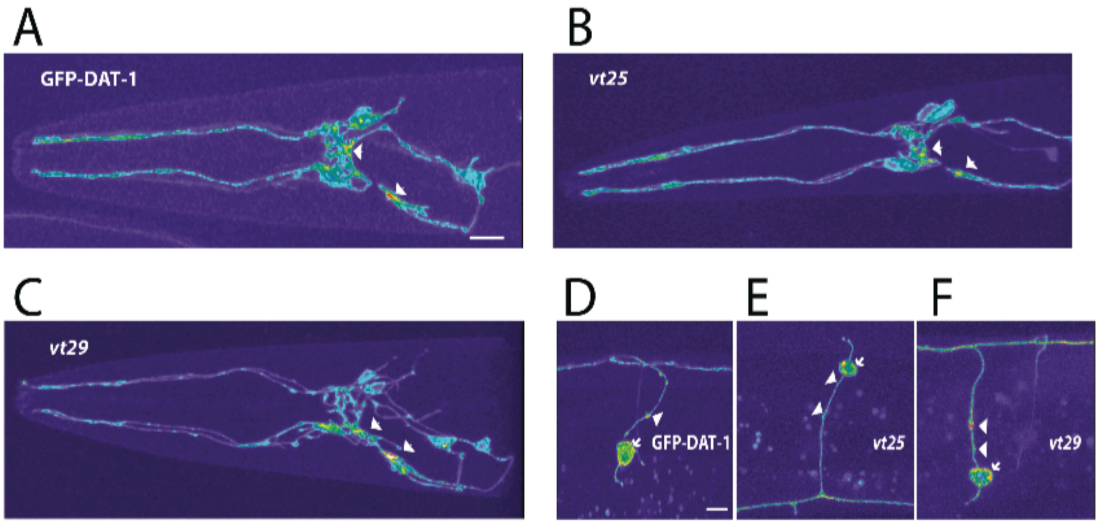

Figure S7 *vt25* and *vt29* do not alter GFP-DAT somatic export: Expression pattern of p_{dat-1}::GFP:DAT-1(*vtIs18)* in the head(CEP and ADE neurons, **A,B + C**) and posterior(PDE, **D,E + F**) in WT, *vt25* and *vt29* were analyzed by confocal microscopy. The expression pattern of GFP:DAT-1 is not altered on the *vt25* or *vt29* backgrounds, with diffuse expression in DA neuron cell bodies(arrows) and punctate expression visible at terminal regions (arrowheads). Scale bar equal 10 μM.
